# A potassium-chloride co-transporter promotes tumor progression and castration resistance of prostate cancer through m^6^A reader YTHDC1

**DOI:** 10.1038/s41419-022-05544-8

**Published:** 2023-01-06

**Authors:** Shuai Yuan, Shao-Hua He, Lu-Yao Li, Shu Xi, Hong Weng, Jin-Hui Zhang, Dan-Qi Wang, Meng-Meng Guo, Haozhe Zhang, Shuang-Ying Wang, Dao-Jing Ming, Meng-Yang Liu, Hailiang Hu, Xian-Tao Zeng

**Affiliations:** 1 Center for Evidence-Based and Translational Medicine, Zhongnan Hospital of Wuhan University, Wuhan, China; 2 Precision Medicine Center, The Second People’s Hospital of Huaihua, Huaihua, China; 3 Department of Urology, Zhongnan Hospital of Wuhan University, Wuhan, China; 4 School of Clinical Medicine, Henan University, Kaifeng, China; 5 Department of Biochemistry, School of Medicine, Southern University of Science and Technology, Shenzhen, China; 6 Key University Laboratory of Metabolism and Health of Guangdong, Southern University of Science and Technology, Shenzhen, China

**Keywords:** Prostate cancer, Prostate cancer

## Abstract

SLC12A5, a neuron-specific potassium-chloride co-transporter, has been reported to promote tumor progression, however, the underlying mechanism remains unclear. Here we report that SLC12A5 functions as an oncogene to promote tumor progression and castration resistance of prostate cancer through the N6-methyladenosine (m^6^A) reader YTHDC1 and the transcription factor HOXB13. We have shown that the level of SLC12A5 was increased in prostate cancer, in comparison to its normal counterparts, and further elevated in castration-resistant prostate cancer (CRPC). The enhanced expression of *SLC12A5* mRNA was associated with neuroendocrine prostate cancer (NEPC) progression and poor survival in prostate cancer. Furthermore, we demonstrated that SLC12A5 promoted the castration resistance development of prostate cancer in addition to the cell proliferation and migration. Interestingly, SLC12A5 was detected in the cell nucleus and formed a complex with nuclear m^6^A reader YTHDC1, which in turn upregulated HOXB13 to promote the prostate cancer progression. Therefore, our findings reveal a mechanism that how the potassium-chloride cotransporter SLC12A5 promotes the tumor progression and provide a therapeutic opportunity for prostate cancer to apply the neurological disorder drug SLC12A5 inhibitors.

## Introduction

Prostate cancer (PCa) has become the second most common malignant tumors in men worldwide and remains a major global public health burden [1–3]. Androgen deprivation therapy (ADT) is the main systemic therapy for PCa patients. Although most PCa patients are initially responsive to ADT [4, 5], they still progress to castration-resistant prostate cancer (CRPC) after several years of treatment [6]. The subsequent treatment of CRPC with second generation of anti-androgen agents results in diverse resistant phenotypes of prostate cancer, including neuroendocrine prostate cancer (NEPC) [7]. The treatment-driven progression from primary adenocarcinoma to CRPC and/or NEPC is accompanied by a series of genetic and epigenetic changes that are essential for cancer cell growth and metastasis [8]. Therefore, identifying key drivers of prostate cancer progression and therapeutic resistance may provide new treatment strategies for prostate cancer.

SLC12A5 (Solute Carrier Family 12 Member 5) is a neuron specific potassium chloride cotransporter 2 (K^+^/Cl^−^ cotransporter 2, KCC2) that is responsible for the transport of chloride ions from neuronal cells to the outside [9]. SLC12A5 cooperates with NKCC1 (Na-K-Cl cotransporter 1), which is responsible for the transport of chloride ions into the cells, to maintain the homeostasis of chloride ion in neurons [10]. Abnormality of SLC12A5 has been shown to associate with neurological diseases (such as epileptic encephalopathy and Rett syndrome) [11, 12], while the encoded protein plays an important role in regulating morphogenesis of developing neurons and the function of GABAergic synapses through chloride homeostasis [13, 14].

Interestingly, recent studies have revealed a non-neuronal oncogenic function for SLC12A5. SLC12A5 gene was found to be amplified in colorectal cancer by single cell sequencing and this amplification contributed to tumor progression and metastasis of colorectal cancer [15, 16]. Another study found that SLC12A5 promoted invasion and metastasis of bladder cancer through NF-κB/MMP-7 signaling pathway [17]. However, the exact mechanisms that how SLC12A5 promotes the tumor progression remain largely unknown.

The N6-methyladenine (m^6^A) modification of RNA, determined by the dynamic interplay between “writers” (methyltransferases) and “erasers” (demethylases), is the most abundant reversible modification to regulate RNA metabolism, such as transcription, pre-mRNA splicing, RNA degradation, and translation [18]. The m^6^A “readers” bind to m^6^A modified sites and exert the ultimate role of m^6^A in RNA processing [18]. YTHDC1 has been reported as the only YTH domain-containing nuclear m^6^A reader and plays multifaceted roles to determine the fates of RNA including transcriptional activation or silencing, RNA splicing, RNA nuclear export and RNA stability [19]. In this study, we demonstrated that SLC12A5 functions as an oncogenic regulator as well in prostate cancer. Mechanistically, SLC12A5 interacts with the m^6^A reader YTHDC1 in the nucleus and in turn upregulates the transcription factor HOXB13 to promote the tumor progression of prostate cancer.

## Results

### SLC12A5 is significantly up-regulated and associated with progression and poor survival in prostate cancer

**Fig. 1:**
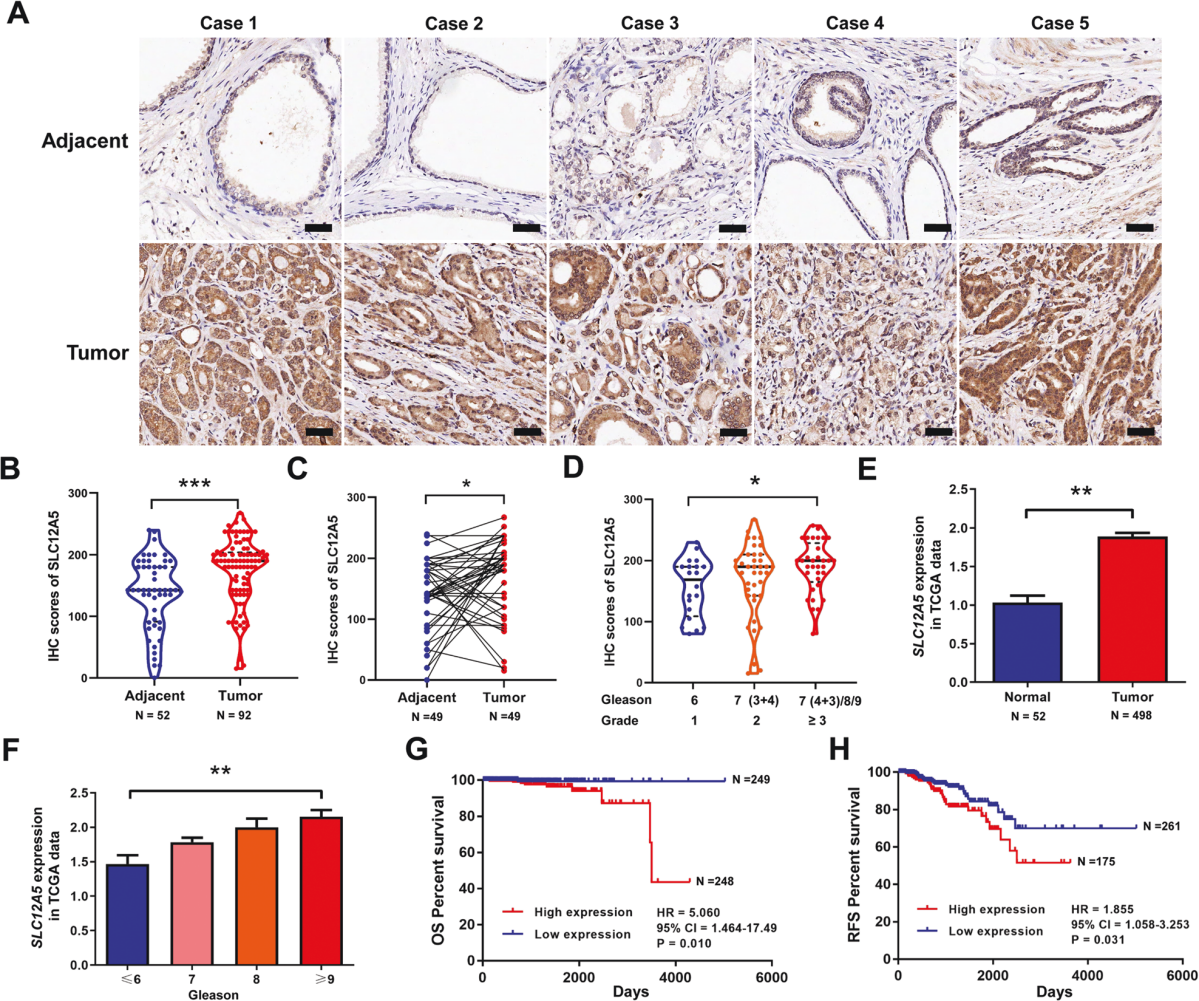
The expression patterns of SLC12A5 in prostate cancer and its clinical significance. **A**, **B** The protein levels of SLC12A5 in 92 prostate tumor tissues and 52 adjacent normal prostate tissues by immunohistochemistry (IHC) on a tissue array. IHC scores of SLC12A5 were compared using Mann–Whitney *U*-test. Scale bars represent 50 μm. **C** IHC scores of SLC12A5 in 49 paired prostate cancer and adjacent normal tissues were compared using Wilcoxon signed-rank test. **D** IHC scores of SLC12A5 in different Gleason score groups were compared using Kruskal-Wallis test. **E** *SLC12A5* mRNA expression in the TCGA prostate cancer (52 normal vs. 498 tumor tissues). Error bars represent the SE of gene expressions. **F** *SLC12A5* mRNA expression levels in patients subgrouped by Gleason score. Error bars represent the SE of gene expressions. **G** Kaplan-Meier curves for overall survival (OS) of prostate cancer patients with high and low expression levels of *SLC12A5* using TCGA data. **H** Kaplan-Meier curves for recurrence free survival (RFS) of prostate cancer patients with high and low expression levels of *SLC12A5* using TCGA data. *P < 0.05; **P < 0.01; ***P < 0.001.

We explored SLC12A5 protein levels in 92 prostate tumor tissues and 52 adjacent normal prostate tissues using immunohistochemical staining. SLC12A5 protein levels were significantly up-regulated in prostate cancer tissues compared with adjacent normal tissues (*P* < 0.01) (Fig. 1A, B). Among these prostate tissues, 49 paired prostate cancer and adjacent normal tissues were available. We compared the SLC12A5 protein levels in 49 paired tissues and confirmed that SLC12A5 protein levels were significantly enhanced in prostate cancer tissues compared with the matched adjacent normal tissues (*P* < 0.01) (Fig. 1C). We next divided these tumor samples into three groups based on Gleason score [Grade 1:6; Grade 2:7 (3 + 4); Grade 3: 7 (4 + 3)/8/9], and found that the SLC12A5 protein levels were significantly increased in higher Gleason score groups (*P* < 0.05) (Fig. 1D). To further validate these results, we subsequently analyzed the TCGA prostate cancer datasets and found that the expression of *SLC12A5* mRNA in prostate tumor tissues was significantly higher than that in normal tissues (*P* < 0.01) (Fig. 1E). The expression levels of *SLC12A5* were also positively correlated with higher Gleason score (Fig. 1F), advanced TNM stage (large tumor size, lymph node spread, distant metastases) and higher PSA levels in TCGA dataset (Supplementary Fig. S1A–D), suggesting a potential oncogenic role of SLC12A5 in prostate cancer progression. We further performed survival analysis using TCGA datasets and found that the high expression of *SLC12A5* was significantly associated with poor overall survival [hazard ratio (HR) = 5.060, 95% confidence interval (CI) = 1.464–17.49, *P* = 0.010)] and recurrence-free survival (HR = 1.855, 95% CI = 1.058–3.253, *P* = 0.031) in patients with prostate cancer (Fig. 1G, H). Moreover, *SLC12A5* expression levels were analyzed by qRT-PCR in five prostate cancer cell lines (LNCaP, C4-2, 22RV-1, DU145 and PC3) that represent different stages of prostate cancer. Compared to the low-grade prostate cancer cell lines (LNCaP and C4-2), the *SLC12A5* expression levels were remarkably higher in the high-grade prostate cancer cell lines (22RV-1, DU145 and PC3) (Supplementary Fig. S1E).

### SLC12A5 promotes prostate cancer progression and therapeutic resistance

**Fig. 2:**
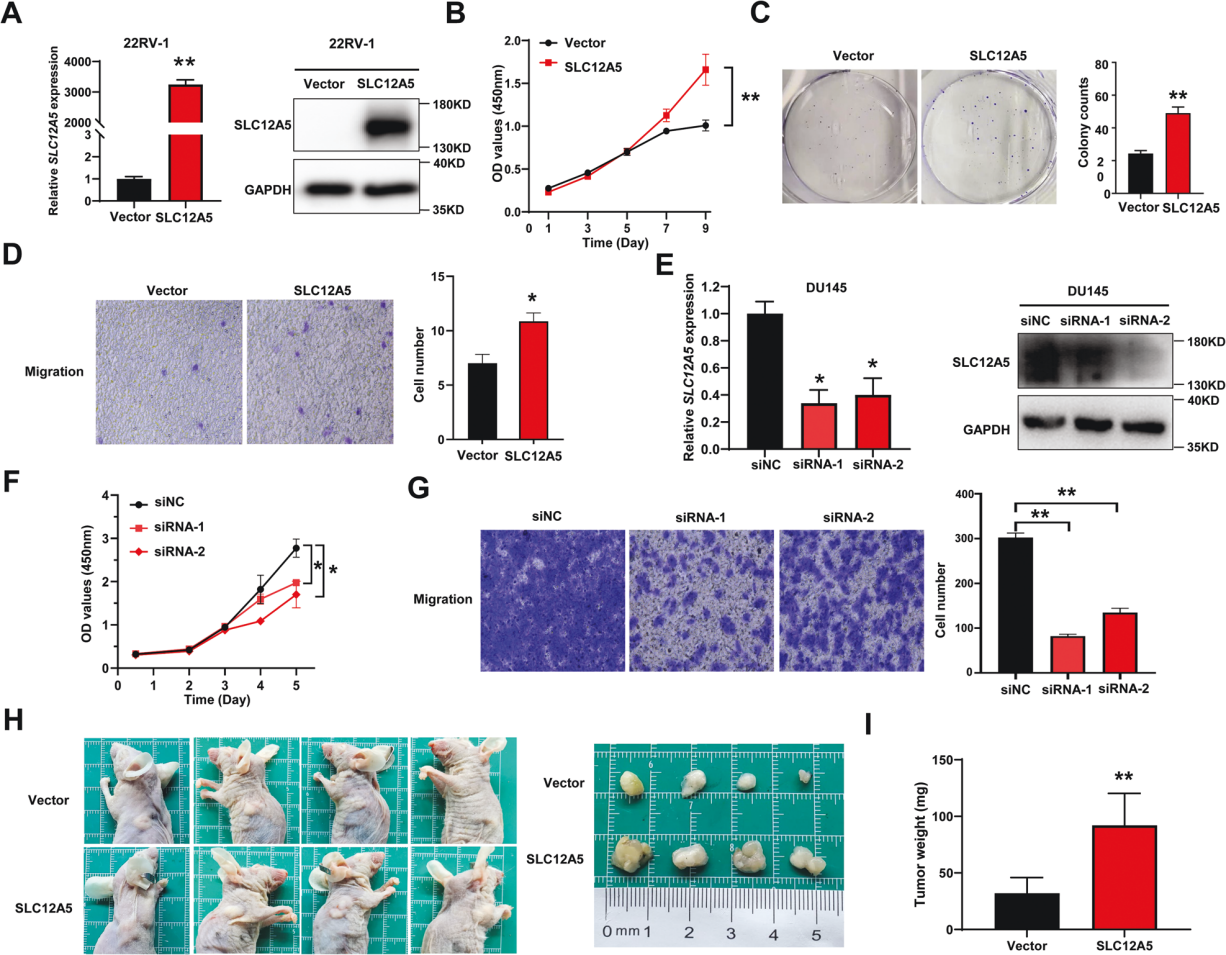
Effects of SLC12A5 on prostate cancer cell proliferation and migration. **A** Exogenous overexpression of SLC12A5 was identified by qRT-PCR and western blot in 22RV-1 cells. **B** Cell proliferation curves were detected by CCK-8 assays after overexpression of SLC12A5 in 22RV-1 cells. **C** Colony formation of 22RV-1 cells after overexpression of SLC12A5 were detected. **D** Migration of 22RV-1 cells after overexpression of SLC12A5 were detected by transwell assays. Magnification, ×200. **E** Knockdown of SLC12A5 by siRNAs were identified by qRT-PCR and western blot in DU145 cells. **F** Cell proliferation curves were detected by CCK-8 assays after knockdown of SLC12A5 in DU145 cells. **G** Migration of DU145 cells after knockdown of SLC12A5 were detected by transwell assays. Magnification, ×200. **H** Subcutaneous xenograft tumors were dissected and photographed. **I** Tumor weight from the SLC12A5 and vector control groups. *P < 0.05; **P < 0.01.

To probe the biological functions of SLC12A5 in prostate cancer, we first established SLC12A5-overexpressing stable cell lines by transfecting Flag-tagged SLC12A5 fusion vector into 22RV-1 and C4-2 cells (Fig. 2A, Supplementary Fig. S2A). The overexpression of SLC12A5 significantly increased the proliferation and colony formation of both 22RV-1 and C4-2 cell lines (*P* < 0.01) (Fig. 2B, C; Supplementary Fig. S2B), in consistent with the previous observation in colorectal cancer (Ref. [16]). Furthermore, SLC12A5 overexpression significantly promoted the migration of 22RV-1 cells (*P* < 0.01) as well (Fig. 2D). In the other hand, knocking down SLC12A5 in DU145 and PC3 cells by small interfering RNAs (siRNAs) significantly decreased cell proliferation (Fig. 2E, F; Supplementary Fig. S2C–E) and cell migration (Fig. 2G, Supplementary Fig. S2F), corroborating with the findings in colorectal cancer (Ref. [16]) and suggesting SLC12A5 as an oncogene in prostate cancer as well. We further confirmed that SLC12A5 promoted tumorigenesis in vivo with subcutaneous xenograft tumor models wherein stably overexpressing SLC12A5 in 22RV-1 cells significantly increased the growth of xenograft tumors (Fig. 2H, I).

**Fig. 3:**
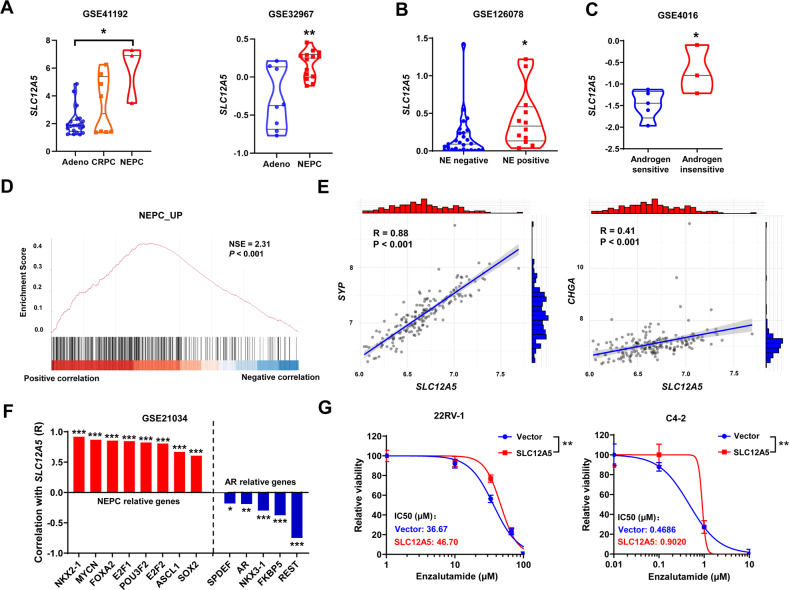
The role of SLC12A5 in castration resistance and neuroendocrine differentiation of prostate cancer. **A** *SLC12A5* mRNA expression patterns in CRPC and NEPC tissues. **B** The expression levels of *SLC12A5* in the NE positive tumor tissues and NE negative tissues in GSE126078 dataset. **C** The expression levels of *SLC12A5* in androgen-insensitive prostate cancer cell lines and androgen-sensitive prostate cancer cell lines in GSE4016 dataset. **D** GSEA showed that *SLC12A5* expression was positively correlated with genes that are upregulated in NEPC using GSE21034 dataset. **E** The correlations of *SLC12A5* with NEPC markers (SYP and CHGA) in GSE21034 dataset. **F** The correlations of *SLC12A5* with NEPC relative genes (*NKX2-1*, *MYCN*, *FOXA2*, *E2F1*, *POU3F2*, *E2F2*, *ASCL1*, *SOX2*) and AR relative genes (*AR, SPDEF, NKX3-1, FKBP5, REST*) in GSE21034 dataset. **G** The effect of SLC12A5 overexpression on the enzalutamide resistance in 22RV-1 and C4-2 cells. IC50 curves and values were generated using Graphpad Prism 8.0. The differences in viability between two groups were evaluated using two-way analysis of variance (ANOVA). *P < 0.05; **P < 0.01; ***P < 0.001.

Next, we assessed whether the overexpression of SLC12A5 in prostate cancer contributed to its castration resistance development. Neuroendocrine differentiation is thought to be one of cellular mechanisms account for the castration resistance. When evaluating the datasets of CRPC and NEPC, we found that *SLC12A5* expression was significantly upregulated in NEPC and CRPC compared with prostate adenocarcinoma samples (Fig. 3A). The expression of *SLC12A5* was further found significantly higher in the NE positive tumor tissues than that in the NE negative tissues (*P* < 0.05) (GSE126078, Fig. 3B) as well as significantly higher in androgen-insensitive prostate cancer cell lines compared to androgen-sensitive prostate cancer cell lines (*P* < 0.05) (GSE4016, Fig. 3C). Gene set enrichment analysis (GSEA) of genes correlated with *SLC12A5* using GSE21034 showed that *SLC12A5* expression was positively correlated with genes that are upregulated in NEPC (Fig. 3D), which includes the NEPC markers such as *SYP* and *CHGA* (Fig. 3E). Besides the NEPC markers, *SLC12A5* was also positively correlated with genes that drive neuroendocrine plasticity in prostate cancer progression such as *NKX2-1*, *MYCN*, *FOXA2*, *E2F1*, *POU3F2* *(BRN2)*, *E2F2*, *ASCL1* and *SOX2*, whereas negatively correlated with AR, androgen-responsive genes (*SPDEF*, *NKX3-1*, and *FKBP5*) and NEPC repressor *REST* (Fig. 3F). To directly confirm whether SLC12A5 contributed to the castration resistance, we overexpressed SLC12A5 in C4-2 and 22RV-1 cells, and found that SLC12A5 overexpression significantly enhanced the resistance of 22RV-1 and C4-2 cells to enzalutamide (Fig. 3G). Taken together, these data suggested that SLC12A5 overexpression could lead to castration resistance and associate with neuroendocrine differentiation in prostate cancer.

### SLC12A5 protein can be detected in the cell nucleus

**Fig. 4:**
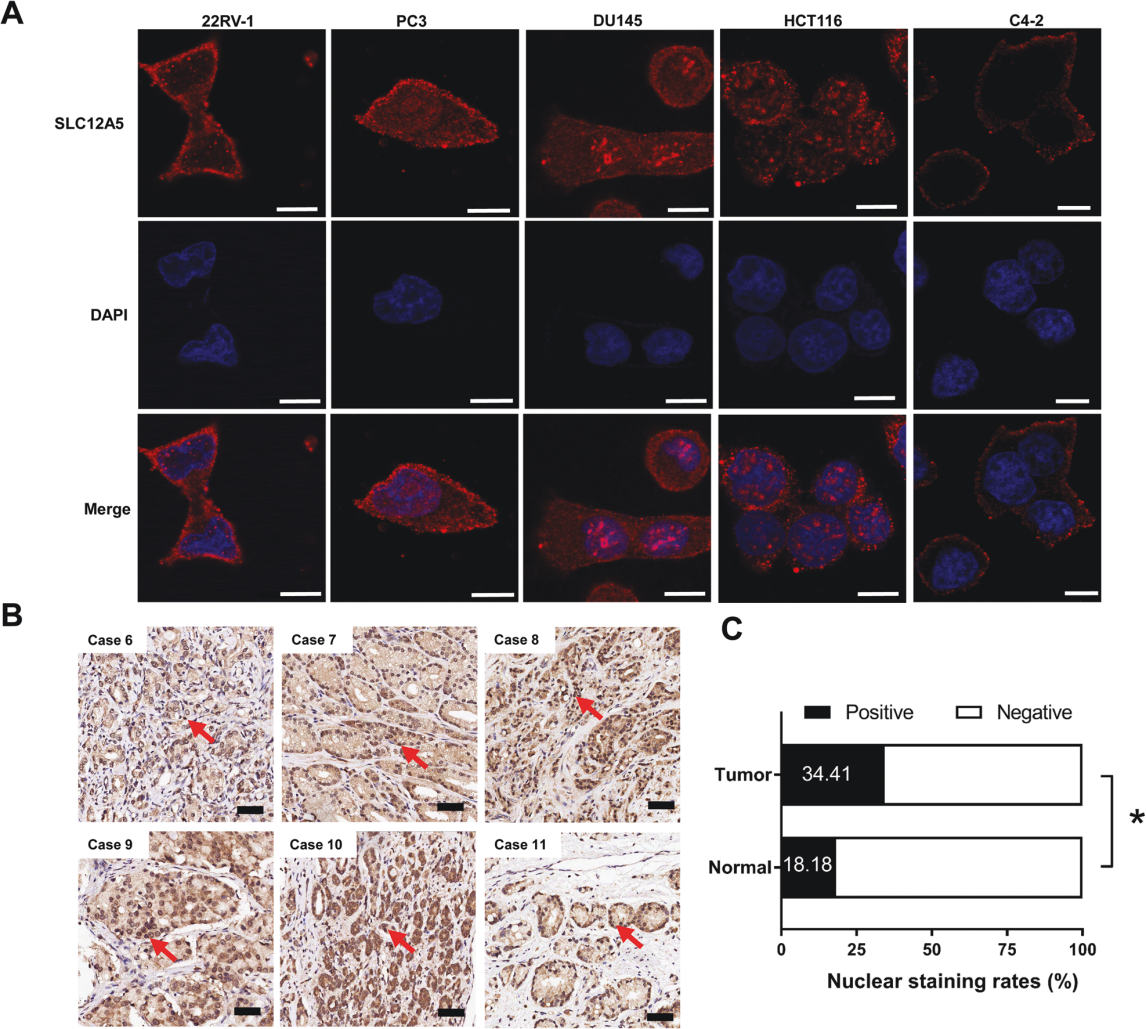
SLC12A5 protein can be detected in the cell nucleus. **A** Immunofluorescence staining was performed to clarify the localization of SLC12A5 protein in SLC12A5 highly expressing prostate cancer cells (22RV-1, PC3 and DU145). Colorectal cancer HCT116 cell line was used as a positive control for nuclear localization and C4-2 cell line was used as a negative control for nuclear localization. Scale bars represent 10 μm. **B** Immunohistochemical staining of prostate cancer tumor tissues (Case 6-Case 11) showed SLC12A5 protein was partly localized in the nucleus. Scale bars represent 50 μm. **C** The positive nuclear staining rates of SLC12A5 protein were compared using χ^2^ (chi-square) test. *P < 0.05.

It is widely recognized that SLC12A5 is a neuron specific membrane protein, which is responsible for the maintenance of the low intracellular Cl^−^ concentration. Therefore it is assumed that SLC12A5 should be localized in the cell membrane. Immunofluorescence staining was then performed to clarify the localization of SLC12A5 protein in SLC12A5 highly expressed prostate cancer cell lines. Surprisingly, we found that SLC12A5 protein was expressed not only in the cell membrane, but also in the nucleus in 22RV-1, PC3 and DU145 cells (Fig. 4A), which is consistent with the observation in colorectal cancer cells (Ref. [16]). We also performed the nucleus and cytoplasm separation assay to confirm the localization of SLC12A5 in nucleus. We found that SLC12A5 protein can be detected in both cytoplasmic and nuclear fractions of 22RV-1, PC3, DU145 and HCT116 (positive control cell line) cells, but not in the nuclear fraction of C4-2 cell (negative control cell line) (Supplementary Fig. S3A). The nuclear localization of SLC12A5 is further confirmed by immunohistochemical staining in prostate cancer tumor tissues as well (Fig. 4B, Supplementary Fig. S3B). Most SLC12A5 protein was localized in the cell surface but some were in the nucleus and the positive nuclear staining rates of SLC12A5 protein were significantly higher in tumor tissue than that in the adjacent normal prostate tissues (Fig. 4C).

### SLC12A5 plays a tumor-promoting role partially depending on YTHDC1 and HOXB13

**Fig. 5:**
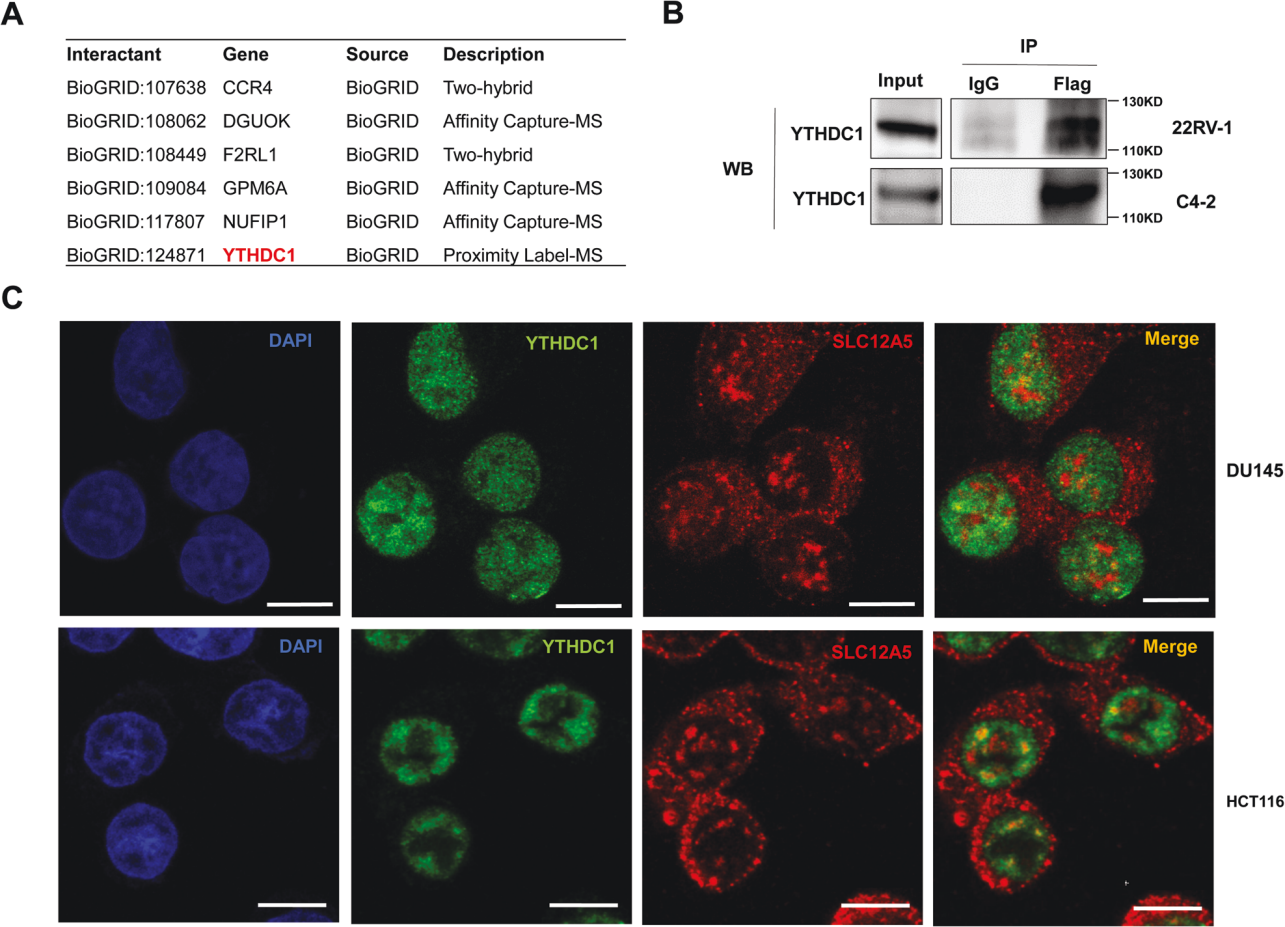
SLC12A5 protein binds to YTHDC1 in the nucleus. **A** The protein interactions of SLC12A5 were predicted using BioGRID. **B** The binding effects of SLC12A5 and YTHDC1 were detected by Co-IP assay in 22RV-1 and C4-2 cells transfected with Flag-tagged SLC12A5 fusion vector. **C** Immunofluorescence staining was performed to clarify the binding effects and co-localization of endogenous SLC12A5 and YTHDC1 in SLC12A5 high-expressing prostate and colorectal cancer cells. Scale bars represent 10 μm.

To explore the potential mechanism by which SLC12A5 contributes to the progression and therapeutic resistance of prostate cancer, a BioGRID (biomedical interaction repository with data compiled through comprehensive curation efforts) analysis was used to seek the SLC12A5 interacting proteins. We identified 6 SLC12A5 interacting proteins, including CCR4, DGUOK, F2RL1, GPM6A, NUFIP1 and YTHDC1 (Fig. 5A, Supplementary Fig. S4A). Interestingly, we also identified an intrinsically disordered region (IDR) in SLC12A5 protein using ESpritz (Supplementary Fig. S4B). Recent studies has highlighted that intrinsically disordered proteins could bind with each other to form well-defined 3D structures to play diverse roles in cell signaling [20, 21]. Then, we further predicted the IDR of the 6 proteins (CCR4, DGUOK, F2RL1, GPM6A, NUFIP1 and YTHDC1) and found that YTHDC1 has more IDRs than other proteins (Supplementary Fig. S4B), suggesting that YTHDC1 might be more likely to interact with the IDR of SLC12A5 protein. Since YTHDC1 carries out its functions mainly in nucleus while SLC12A5 has a nuclear localization as well, the interaction between SLC12A5 and YTHDC1 may suggest that YTHDC1 is likely able to mediate the SLC12A5 functions. Therefore, we first conducted a co-immunoprecipitation assay to verify whether SLC12A5 could interact with YTHDC1 in prostate cancer cells. Flag tagged SLC12A5 constructs were transfected into 22RV-1 and C4-2 cells (Supplementary Fig. S4C). As shown in Fig. 5B, Flag-SLC12A5 was able to bind with YTHDC1 in prostate cancer cells. To further determine whether the interaction between endogenous SLC12A5 and YTHDC1 occurs in the cell nucleus, we conducted an immunofluorescence staining and found that endogenous SLC12A5 co-localized with YTHDC1 in the cell nucleus in SLC12A5 high-expressing prostate and colorectal cancer cells (Fig. 5C). We also examined the mutual regulatory effect between SLC12A5 and YTHDC1. We found that overexpression of SLC12A5 in prostate cancer cells did not remarkably alter the protein levels of YTHDC1 (Supplementary Fig. S4C) and knockdown of YTHDC1 in 22RV-1 cells did not change the SLC12A5 protein levels (Supplementary Fig. S4D) either, ruling out the possible mutual regulatory effect between SLC12A5 and YTHDC1 at the protein level.

**Fig. 6:**
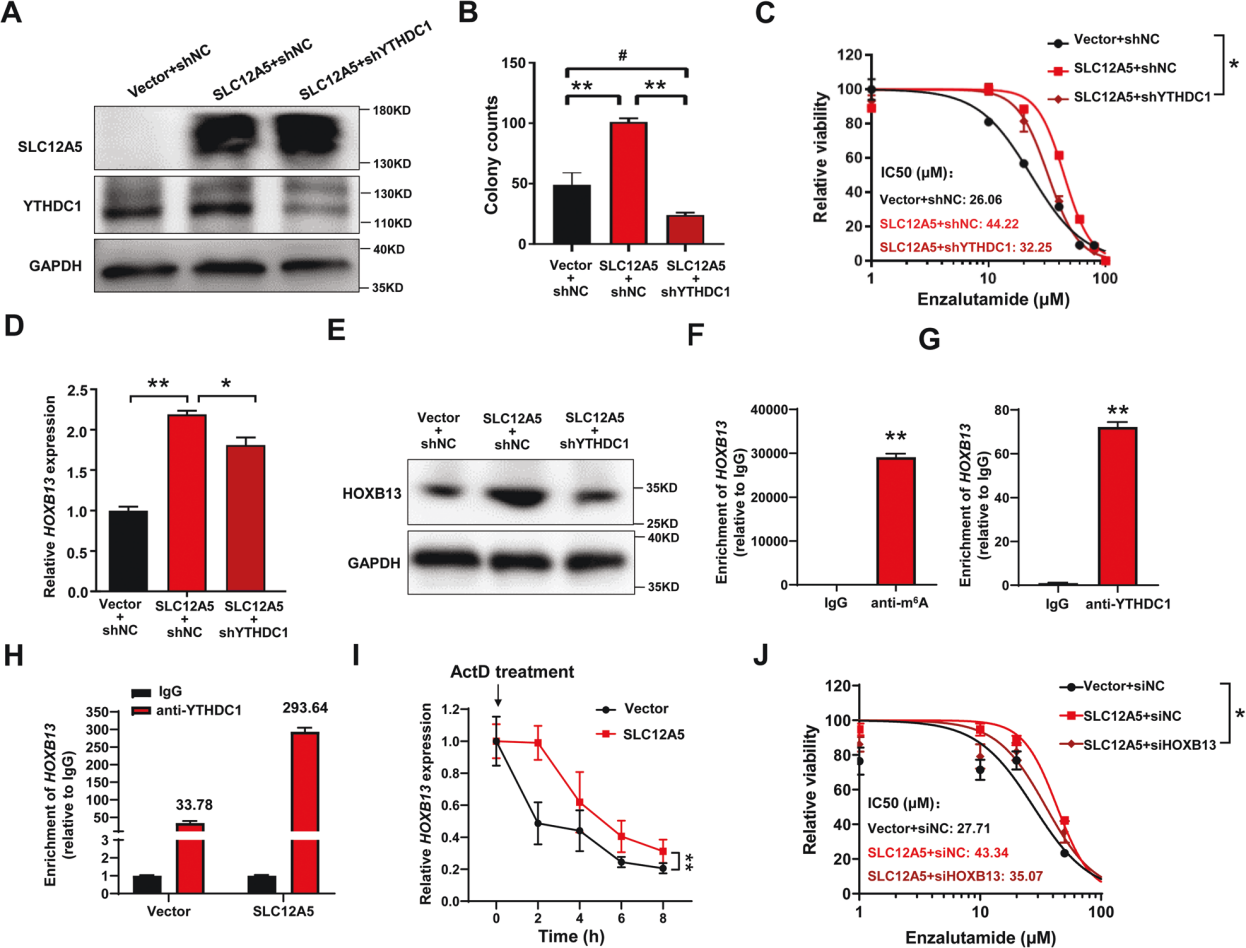
SLC12A5 plays a tumor-promoting role partially depending on YTHDC1 and HOXB13. **A** Knockdown of YTHDC1 was identified by WB in 22RV-1 cells which already stably overexpressed SLC12A5. **B** Colony formation assay was conducted to clarify the rescue effect of YTHDC1 on the cell proliferation in 22RV-1 cells. **C** Enzalutamide treatment was conducted to clarify the rescue effect of YTHDC1 on the castration resistance in 22RV-1 cells. IC50 curves and values were generated using Graphpad Prism 8.0. The differences in viability between three groups were evaluated using two-way analysis of variance (ANOVA). **D** The expression levels of *HOXB13* mRNA were detected by qRT-PCR to clarify the rescue effect of YTHDC1 on the target gene expression in 22RV-1 cells. **E** The protein levels of HOXB13 were detected by western blot to clarify the rescue effect of YTHDC1 in 22RV-1 cells. **F** The m^6^A RNA modification of *HOXB13* mRNA was detected by MeRIP assay in 22RV-1 cells. **G** The binding effects of YTHDC1 to *HOXB13* mRNA was detected by RIP assay in 22RV-1 cells. **H** The binding effects of YTHDC1 to *HOXB13* mRNA was detected by RIP assay after overexpression of SLC12A5 in 22RV-1 cells. **I** Stability of *HOXB13* mRNA is measured by qRT-PCR relative to time 0 h after blocking new RNA synthesis with Actinomycin D (10 μg/mL) in SLC12A5-overexpressing 22RV-1 cells and control cells. **J** Enzalutamide treatment was conducted to clarify the rescue effect of HOXB13 knockdown on the castration resistance in 22RV-1 cells. IC50 curves and values were generated using Graphpad Prism 8.0. The differences in viability between three groups were evaluated using two-way analysis of variance (ANOVA). *P < 0.05; **P < 0.01; ^#^*P* > 0.05.

We next determined whether YTHDC1 can mediate the SLC12A5’s oncogenic function in prostate cancer cells. We knocked down the YTHDC1 in 22RV-1 cells which already stably overexpressed SLC12A5 (Fig. 6A) and found that knockdown of YTHDC1 abrogated the promotive effect of SLC12A5 on the cell colony formation (Fig. 6B, Supplementary Fig. S5A) as well as partially rescued the enzalutamide sensitivity of SLC12A5-overexpressing 22RV-1 cells (Fig. 6C). Taken together, these results indicated that SLC12A5 interacted with YTHDC1 to form a SLC12A5-YTHDC1 complex in the cell nucleus, thus mediated the progression of prostate cancer.

As SLC12A5 is associated with the neuroendocrine differentiation of prostate cancer (Fig. 3) while YTHDC1 regulates gene expression in an m^6^A-dependent manner [19]. We selected 12 genes, which have been demonstrated to be required for the enzalutamide resistance and neuroendocrine differentiation of prostate cancer, and determined whether their expression are affected by the SLC12A5/YTHDC1 complex. We found that *HOXB13* mRNA was up-regulated in SLC12A5-overexpressing 22RV-1 cells (Supplementary Fig. S5B) and this upregulation was partially abrogated by knocking down YTHDC1 in the SLC12A5-overexpressing 22RV-1 cells (Fig. 6D, E), suggesting that HOXB13 was the target gene of SLC12A5/YTHDC1 complex in prostate cancer. By using SRAMP prediction program (http://www.cuilab.cn/sramp), a mammalian m^6^A sites predictor, we identified *HOXB13* mRNA could be modified by m^6^A with high confidence (Supplementary Fig. S5C). The methylated RNA immunoprecipitation (MeRIP)-qPCR assays confirmed that *HOXB13* mRNA was indeed modified by m^6^A in 22RV-1 cells (Fig. 6F). Our RNA immunoprecipitation (RIP) assay further confirmed HOXB13 mRNA could also be recognized by YTHDC1 and bound to YTHDC1 (Fig. 6G). The RIP assay further showed that overexpression of SLC12A5 increased the binding effect of YTHDC1 to *HOXB13* mRNA (Fig. 6H). YTHDC1 was recently reported to maintain the mRNA stability of its target genes [22]. To further test whether the stability of *HOXB13* mRNA was affected by SLC12A5/YTHDC1 complex, Actinomycin D (ActD) was used to block new RNA synthesis and then the degradation rate of *HOXB13* mRNA was measured by qRT-PCR in 22RV-1 cells. We found that overexpression of SLC12A5 significantly promoted the stability of *HOXB13* mRNA in 22RV-1 cells (Fig. 6I). Functionally, knocking down the HOXB13 using siRNAs in SLC12A5-overexpressing 22RV-1 cells partially rescued effect of SLC12A5 on the enzalutamide resistance (Fig. 6J). Thus, HOXB13 is the target gene of SLC12A5/YTHDC1 complex in the nucleus that mediates the pro-tumor function of SLC12A5 in prostate cancer.

## Discussion

**Fig. 7:**
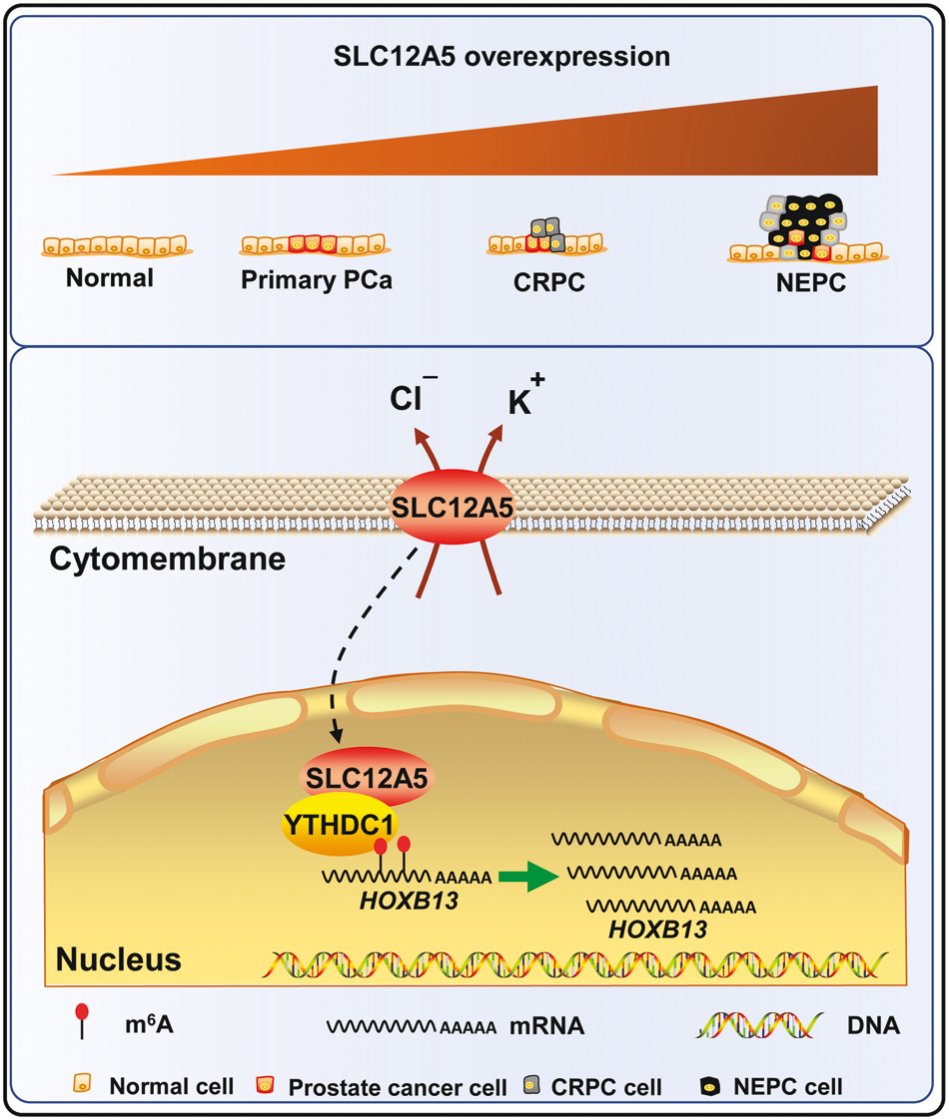
A working model of SLC12A5 in prostate cancer. SLC12A5 is gradually increased during the tumorigenesis and progression of prostate cancer. Mechanistically, the nuclear SLC12A5 binds with nuclear m^6^A reader YTHDC1 to form the SLC12A5-YTHDC1 complex and regulates the HOXB13, which mediates the tumor promoting role of SLC12A5 in prostate cancer.

In this study, we have shown that SLC12A5 increased over the prostate cancer progression and was positively correlated with higher Gleason score, advanced TNM stage, and poor survivals in prostate cancer patients. Furthermore, we demonstrated that SLC12A5 can function as an oncogene to promote cell proliferation and migration, thus leading to the castration resistance and neuroendocrine differentiation of prostate cancer. Mechanistically, SLC12A5 moves to the nucleus and binds with the nuclear m^6^A reader YTHDC1 to form the SLC12A5/YTHDC1 complex and regulates the HOXB13 (Fig. 7).

Abnormal activation of AR pathway is an important feature of tumorigenesis and progression in prostate cancer [23]. ADT by chemical (luteinizing hormone-releasing hormone agonist or antagonist) or surgical castration is becoming the first-line treatment for patients with locally advanced or extensive metastatic prostate cancer [4]. Even though patients with prostate cancer could benefit from ADT, most patients will progress to CRPC. To further inhibit the AR pathway in CRPC, the second generation of AR pathway inhibitors (ARPIs), such as abiraterone and enzalutamide, were developed [24, 25]. Unfortunately, the treatment of these ARPIs will ultimately result in drug resistance, and patients with CRPC could progresses to therapy-induced NEPC (t-NEPC), which is independent on AR signaling and leads to treatment failure [26]. Complex genomic changes underlying and regulating the progression of prostate cancer to CRPC and t-NEPC have been reported, such as deletion of tumor suppressors (RB1, TP53 and PTEN), activation of several transcription factors (BRN2, ASCL1, SOX2, N-Myc, and E2F1), and epigenetic remodeling (EZH2 and LSD1) [7, 26]. In the present study, we demonstrated that expression of SLC12A5 was gradually increased in prostate adenocarcinoma, CRPC and NEPC tissues. Correspondingly, the expression of *SLC12A5* mRNA was positively correlated with CRPC and NEPC driver genes, including BRN2, ASCL1, SOX2, N-Myc, and E2F1. Functionally, SLC12A5 increased the enzalutamide resistance in prostate cancer cells. Our data suggested that SLC12A5 was a novel player in the steps of CRPC progression and neuroendocrine differentiation in prostate cancer.

In previous studies, SLC12A5 has been identified as a membrane protein that plays a key role in K-Cl transporting. However, SLC12A5 was also found to be expressed in the nucleus of prostate cancer cell lines and tissues, which was in consistent with the previous observation in colorectal cancer [16]. Encouraged by these results, we hypothesized that SLC12A5 may perform its biological function by entering the nucleus and play a regulatory role by interacting with other molecules. Our co-immunoprecipitation and immunofluorescence cell staining assays confirmed that SLC12A5 directly bound to YTHDC1 in the nucleus of prostate cancer cells (the co-localization of SLC12A5 and YTHDC1 in the nucleus was also found in colorectal cancer cells). YTHDC1 is a nuclear m^6^A reader which recognizes m^6^A modified pre-mRNAs and mRNAs, and regulates gene expression at the transcriptional or post-transcriptional levels [27]. YTHDC1 has been identified as a critical regulator of tumorigenesis, drug sensitivity, and resistance in several cancers [28–30]. In the present study, we demonstrated YTHDC1 could mediate the tumorigenesis and castration resistance promoting role of SLC12A5 by forming a SLC12A5-YTHDC1 complex in prostate cancer.

To further provide an insight into the downstream molecular events. We selected several oncogenes that promote the malignant transformation of prostate cancer, especially several transcription factors such as BRN2 and HOXB13 [31, 32]. We revealed HOXB13 was the target gene of SLC12A5-YTHDC1 complex. Correspondingly, *HOXB13* mRNA was modified by m^6^A and could be recognized by nuclear m^6^A reader YTHDC1, which supported the regulating effects of SLC12A5-YTHDC1 complex. HOXB13 is a member of the HOX family. Recent studies have found HOX proteins not only have important roles in development and organogenesis, but also contribute to the tumorigenesis and progression by regulating cell proliferation, cell cycle, apoptosis, cell differentiation, and cell migration [33]. In prostate cancer, HOXB13 has been proved as overexpressed in prostate cancer, and showed a higher degree of overexpression in CRPC tissues relative to prostate adenocarcinoma tissues [34]. The germline mutations of HOXB13 has strong associations with prostate cancer risk [35–38], marking it as a suitable specific biomarker for prostate cancer. Functionally, HOXB13 plays oncogenic roles in promoting progression and castration resistance of prostate cancer. Mechanistically, HOXB13 could physically interact with both AR and AR-V7 and co-localizes with AR (or AR-V7) binding sites to enhance the downstream target genes [39, 40].

We have demonstrated HOXB13 as a target gene of SLC12A5-YTHDC1 complex, supporting the oncogenic role of SLC12A5 in prostate cancer. However, it is still remains unclear how SLC12A5-YTHDC1 complex regulates the gene expression. Recently, liquid-liquid phase separation (LLPS) has been proposed to be an important mechanisms for gene expression control by assembling intracellular structures [41]. In the nucleus, such phase separation is involved in gene expression regulation in a variety of ways such as heterochromatin formation, super-enhancers formation [42–44]. YTHDC1 is also involved in phase separation to control gene expression by facilitating transcriptional condensate formation [45]. Cheng et al. has also demonstrated YTHDC1 could undergo phase-separation and form nuclear YTHDC1-m^6^A condensates that maintains mRNA stability and control cancer cell survival and differentiation [46]. Considering that both SLC12A5 and YTHDC1 contain IDRs, which can mediate the formation of liquid–liquid phase separation, we hypothesized that membrane protein SLC12A5 may directly or indirectly affect the phase-separations of YTHDC1 to regulate biological processes in the nucleus. Notably, this hypothesis should be empirically tested, and further studies are required to address this regulatory mechanism.

Our study has several potential novelties and clinical implications. (1) SLC12A5 is a traditional membrane protein, which is believed to mediate K-Cl transport in the cell membrane and maintain intracellular chloride homeostasis to mediate the biological functions. However, our study demonstrates that SLC12A5 is also expressed in the nucleus and exerts a tumor-promoting function in the nucleus. The different subcellular compartments of SLC12A5 may play different roles in a context-dependent manner. Further studies are needed to distinguish the biological functions of these two subcellular compartments, and to address the underlying nuclear translocation mechanisms of SLC12A5. (2) The m^6^A modification of mRNA is now recognized as a key regulator of gene expression and YTHDC1-mediated nuclear m^*6*^A recognition to exert multifaceted effects [19]. Our data reveal SLC12A5 could bind with YTHDC1 to form a SLC12A5-YTHDC1 complex to regulate prostate cancer specific transcription factor HOXB13 in an m^6^A-dependent manner, uncovering a novel mechanism about how SLC12A5 promotes tumor progression. (3) SLC12A5 is a neuron-specific chloride-extruder that shapes cortical development and plays an important role in neurological disorders (such as epilepsy) [47], and we find that SLC12A5 also acts as an oncogene and is associated with CRPC/NEPC progression in prostate cancer. Our study provides a possibility that drugs targeting SLC12A5 in neurological disorders may also be effective in prostate cancer.

In conclusion, our study demonstrates SLC12A5 is gradually increased during the tumorigenesis and progression of prostate cancer. SLC12A5 acts as a novel functional oncogene by promoting prostate cancer cell proliferation and migration, and conferring castration resistance and neuroendocrine differentiation in prostate cancer. Mechanistically, the nuclear SLC12A5 binds with nuclear m^6^A reader YTHDC1 to form the SLC12A5-YTHDC1 complex and regulates the HOXB13. These findings highlight SLC12A5 as an attractive therapeutic target for prostate cancer.

## Materials and methods

### Cell lines and animals

The prostate cancer cell lines LNCaP, C4-2, 22RV-1, DU145 and PC3 were obtained from the Cell Bank of the Chinese Academy of Science (Shanghai, China) or the American Type Culture Collection (ATCC, Manassas, VA, USA). LNCaP, C4-2, 22RV-1 and PC3 cells were cultured in RPMI-1640 (Gibco, Life Technology, Carlsbad, CA, USA) supplemented with 10% fetal bovine serum (Gibco). DU145 cells were cultured in DMEM (Gibco) supplemented with 10% fetal bovine serum (Gibco). The 6 weeks old male BALB/c-nude mice were purchased from the purchased from Vital River (Charles River China, Beijing, China).

### Tissue microarray (TMA) analysis

Tissue microarrays contained 95 prostate cancer tissues (3 tissues excluded from the analysis due to low tumor-cell content), 52 adjacent non-tumorous prostate tissues and 3 normal prostate tissues were obtained from Shanghai Outdo Biotech Company (Shanghai, China) with the agreement of the patients. IHC staining was performed using a mouse monoclonal antibody against SLC12A5 (Abcam). SLC12A5 immunostaining was scored based on the extent of positive cell staining (0%, 1–25%, 26–50%, 51–75%, 76–100%) and the staining intensity (0, no staining; 1, slight staining; 2, moderate staining; and 3, strong staining). All subjects signed an informed consent form. This study was approved by the Medical Ethical Committee, Zhongnan Hospital of Wuhan University (Ethics number 2020164).

### RNA extraction and qRT-PCR analysis

Total RNA was isolated using the TRIzol reagent (TaKaRa, Dalian, China). Real-time PCR was performed using the SYBR Premix Ex Taq (TaKaRa) following the manufacturer’s instructions. Results were normalized to the expression levels of GAPDH and were calculated using 2^^-(ΔΔCt)^ method. Primers sequences were provided in Supplementary Table S1.

### Plasmid construction and cell transfection

To construct a plasmid expressing SLC12A5, the coding sequence of human SLC12A5 (NM_020708) was synthesized and cloned into the GV492 vector (Genechem, Shanghai, China). Lentivirus is used to pack the plasmid containing the SLC12A5 gene and co-transfected into 293 T cells. After purification of lentivirus and quality inspection, the lentivirus contain SLC12A5 gene were transfected into cells. The stably transfected cells were screened under 2 μg/ml puromycin (Biosharp, China) selection for 1 week and 1 μg/ml puromycin was to maintain selection pressure on stably transfected. For SLC12A5 knockdown, 5 μL siRNA (20 μM, GenePharma, Shanghai, China) were transfected into cells with 5 μL Lipofectamine 2000 (Invitrogen) in 6-well plates. For YTHDC1 knockdown, shRNA and corresponding control lentiviruses were synthesized by GeneChem (Shanghai, China). SLC12A5 siRNA and YTHDC1 shRNA target sequences were shown in Supplementary Table S1.

### Cell proliferation assay

Cells (2 × 10^3^) were seeded in a 96-well plate and cell proliferation was assessed using the cell counting kit-8 (CCK-8, Dojindo Laboratories, Kumamoto, Japan). All of the experiments were performed in triplicate. The cell proliferation curves were plotted using the absorbance at each time point. To observe the inhibition of enzalumatide (MCE), 2000 cells in 96-well plate were treated with the indicated concentrations of drug and then incubated for 72 h. CCK-8 assay was performed to measure cell viability at the indicated time points. IC50 values were calculated using Graphpad Prism.

### Colony formation assay

Cells (*n* = 500) were placed into six-well plates and maintained in media for 2 weeks. The medium was replaced every 3 days. Colonies were fixed with methanol and stained with 0.1% crystal violet (Beyotime Biotechnology, Shanghai, China) for 15 min. The visible colonies were then counted.

### Cell migration assays

Cells (3 × 10^4^) in serum-free media were placed into the upper chamber of an insert (8-μm pore size; Millipore, Billerica, MA, USA). Medium containing 10% fetal bovine serum was added to the lower chamber. After incubation for 24 h, the cells remaining on the upper membrane were removed with cotton wool. Cells that had migrated through the membrane were stained and counted with methanol and 0.1% crystal violet.

### Animal experiments in vivo

For subcutaneous xenograft model, 22RV-1 cells (5.0 × 10^6^) which already stably overexpressed SLC12A5, suspended in 200 μL of 50% Matrigel (Corning, New York, USA), were injected subcutaneously into the both flanks of the four nude mice (left: vector control cells vs. right: SLC12A5-overexpressing 22RV-1 cells). The mice were sacrificed 25 days later, and the subcutaneous tumor were removed for further analysis. All experimental animal procedures were approved by the Animal Ethics Committee of Wuhan University.

### Immunofluorescence cell staining

Cells were grown on confocal petri dish (Biosharp, China), fixed in 4% paraformaldehyde for 15 min, permeabilized by 0.1% Triton X-100, and then blocked with 1% BSA for 30 min. The cells were incubated overnight at 4 °C using mouse anti-SLC12A5 (Abcam), mouse anti-Flag (Sigma), or rabbit anti-YTHDC1 (Abcam). After washing three times, the cells were probed with Alexa-Fluor-488-conjugated goat anti-rabbit-IgG (Invitrogen) or Alexa-Fluor-647-conjugated goat anti-mouse-IgG (Invitrogen) for 1 h at room temperature, followed by nuclear counterstaining with DAPI (Beyotime). Coverslips were observed with a fluorescence microscope (Leica, Germany).

### Western blot and Co-Immunoprecipitation (Co-IP) assays

Western blot (WB) and co-immunoprecipitation (Co-IP) were performed as described previously [48]. The antibodies used in this study were rabbit anti-SLC12A5 (1:1000; Abcam), rabbit anti-YTHDC1 (1:1000; Abcam) and rabbit anti-GAPDH (1:5000; Abcam). For Co-IP assay, 22RV-1 and C4-2 cells with SLC12A5 overexpression were lysed with IP lysis buffer and the co-IP analyses were performed using a Co-Immunoprecipitation Kit (Thermo Fisher Scientific, US) according to the manufacturer’s protocol using mouse anti-Flag (Sigma). Subsequent WB analyses were performed.

### RNA immunoprecipitation (RIP) and m^6^A immunoprecipitation (MeRIP) assays

RIP assay was carried out in 22RV-1 cells using EZ-Magna RIP^TM^ RNA-Binding Protein Immunoprecipitation Kit (Millipore, MA) following the manufacturer’s instructions. In briefly, Magnetic beads pre-coated with 5 μg normal antibodies against YTHDC1 (Abcam) or rabbit IgG (Millipore) were incubated with cell lysates (1 × 10^7^ cells per sample) at 4 °C overnight. Then, the beads containing immunoprecipitated RNA-protein complex were treated with proteinase K (10 mg/mL) to remove proteins. Then RNAs were purified and the expression of *HOXB13* in immunoprecipitated RNAs was assessed by qRT-PCR assay.

To determine the m^6^A levels in *HOXB13* mRNA, the m^6^A immunoprecipitation (MeRIP) assay was performed as described previously [49]. In briefly, 5 μg anti-m^6^A antibody (ABclonal, Wuhan, China) or normal rabbit IgG were bound to magnetic beads (Millipore). A total of 50 μg RNA from 22RV-1 cells was immunoprecipitated using anti-m^6^A antibody in RIP immunoprecipitation buffer (Millipore) overnight at 4 °C. After treating with proteinase K (10 mg/mL), RNA were purified and the expression of *HOXB13* in immunoprecipitated RNAs was assessed by qRT-PCR assay. Primers sequences were provided in Supplementary Table S1.

### Bioinformatics analysis

The Cancer Genome Atlas (TCGA) prostate cancer RNAseq data were used to analyze the expression patterns of *SLC12A5* mRNA in the TCGA prostate. To analyze the prognostic value of *SLC12A5*, the relapse free and overall survival information data from TCGA datasets were also download. To analyze the expression of SLC12A5 in CRPC and NEPC, the GSE41192, GSE32967, GSE21034, GSE126078, and GSE4016 from Gene Expression Omnibus (GEO) were also download.

### Statistical analysis

Statistical analyses were performed with the SPSS 22.0 software (SPSS, Inc., Chicago, IL, USA). Results of gene expression, colony formation, and migration were evaluated using the two-tailed Student’s *t*-test. The cell proliferation curve were evaluated using two-way analysis of variance (ANOVA). IC50 curves and values were generated using Graphpad Prism 8.0. A two-sided *P*-value less than 0.05 was taken as statistically significant.

## Supplementary information

Supplementary table and figures

Original uncropped Western Blots

Reproducibility checklist

## Data Availability

All data needed to evaluate the conclusions are present in the paper. The uncropped western blots are shown in Supplementary Information as an ‘Uncropped Western Blots’ file.
