# Supplementary material for: A potassium-chloride co-transporter promotes tumor progression and castration resistance of prostate cancer through m6A reader YTHDC1
Source: Cell Death Dis. 2023 Jan 6;14(1):7. doi: 10.1038/s41419-022-05544-8 (PMC9822915; doi:10.1038/s41419-022-05544-8)
Supplement: Supplementary file 1 — Supplementary table and figures [file 41419_2022_5544_MOESM1_ESM.doc]

**Supplementary Table S1. Primers** and RNA oligonucleotides sequences used in this study.

| **Gene** | **Sequence (5’-3’)** | |
| --- | --- | --- |
| ***SLC12A5*** | Forward: | GCAGGAGCCATGTACATCCT |
|  | Reverse: | CCATGCAGGTGAGCACACA |
| ***YTHDC1*** | Forward: | TCAGGAGTTCGCCGAGATGTGT |
|  | Reverse | AGGATGGTGTGGAGGTTGTTCC |
| ***HOXB13*** | Forward: | ACAGAACCCACCAGGTCCCTTT |
|  | Reverse: | TACGGAATGCGTTTCTTGCGGC |
| ***BRN2*** | Forward: | GTGTTCTCGCAGACCACCATCT |
|  | Reverse: | GCTGCGATCTTGTCTATGCTCG |
| ***CCND2*** | Forward: | GAGAAGCTGTCTCTGATCCGCA |
|  | Reverse: | CTTCCAGTTGCGATCATCGACG |
| ***E2F3*** | Forward: | AGCGGTCATCAGTACCTCTCAG |
|  | Reverse: | TGGTGAGCAGACCAAGAGACGT |
| ***EGF*** | Forward: | TGCGATGCCAAGCAGTCTGTGA |
| ***FGFR1*** | Reverse: | GCATAGCCCAATCTGAGAACCAC |
|  | Forward: | GCACATCCAGTGGCTAAAGCAC |
| ***FOXA1*** | Reverse: | AGCACCTCCATCTCTTTGTCGG |
|  | Forward: | GCAATACTCGCCTTACGGCTCT |
|  | Reverse: | GGGTCTGGAATACACACCTTGG |
| ***JARID2*** | Forward: | GGACAAAGGCGTCCTCAATGAC |
|  | Reverse: | GCAGGCTCCTTGCTGAAACACA |
| ***KLF4*** | Forward: | CATCTCAAGGCACACCTGCGAA |
|  | Reverse: | TCGGTCGCATTTTTGGCACTGG |
| ***SMAD2*** | Forward: | GGGTTTTGAAGCCGTCTATCAGC |
|  | Reverse: | CCAACCACTGTAGAGGTCCATTC |
| ***TCF4*** | Forward: | GCCTCTTCACAGTAGTGCCATG |
|  | Reverse: | GCTGGTTTGGAGGAAGGATAGC |
| ***ZFHX3*** | Forward: | ACACCAACAGCCTGGAGAAGCT |
|  | Reverse: | CACAGAACGCAGTGGTAGTAGC |
| ***GAPDH*** | Forward: | CCATGGAGAAGGCTGGGG |
|  | Reverse | CAAAGTTGTCATGGATGACC |
| **SLC12A5 siRNA-1** | Target seq | GGCTCAATCCGGAGAAAGA |
| **SLC12A5 siRNA-2** | Target seq | CCTTATGTCTTCAGTGATA |
| **siHOXB13** | Target seq | CCCGTGCCTTATGGTTACT |
| **YTHDC1 shRNA** | Target seq | TGGATTTGCAGGCGTGAATTA |


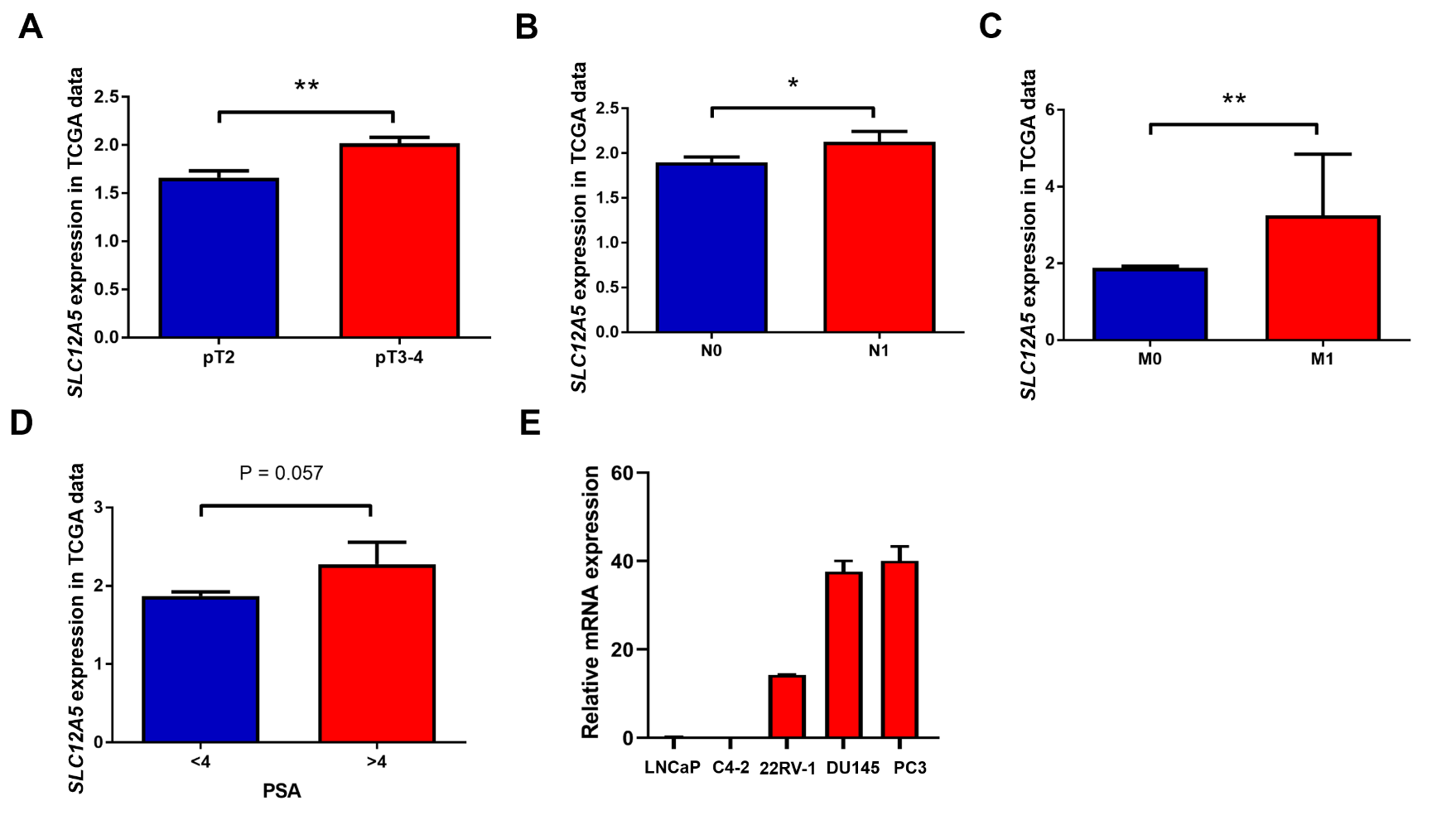


**Supplementary Fig. S1. The expression patterns of SLC12A5 in prostate cancer.** (A) *SLC12A5* mRNA expression levels in patients subgrouped by tumor size in TCGA data. (B) *SLC12A5* mRNA expression levels in patients subgrouped by lymph node spread in TCGA data. (C) *SLC12A5* mRNA expression levels in patients subgrouped by distant metastases in TCGA data. (D) *SLC12A5* mRNA expression levels in patients subgrouped by PSA level in TCGA data. (E) The expression levels of *SLC12A5* in five prostate cancer cell lines LNCaP, C4-2, 22RV-1, DU145 and PC3. *, *P* < 0.05; **, *P* < 0.01.


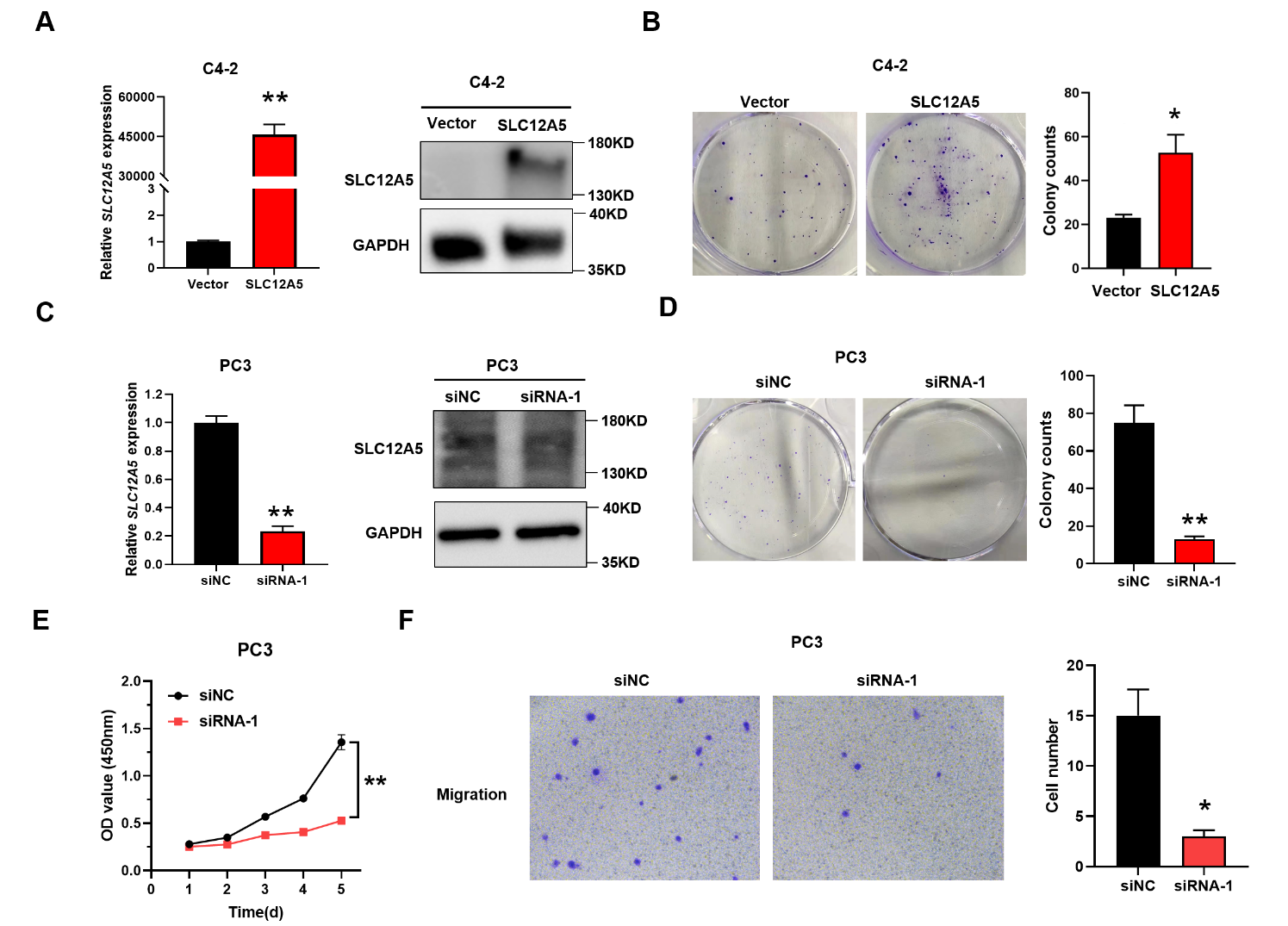


**Supplementary Fig. S2. Effects of SLC12A5 on prostate cancer cell proliferation and migration *in vitro*.** (A) Exogenous overexpression of SLC12A5 was identified by qRT-PCR and western blot in C4-2 cells. (B) Colony formation of C4-2 cells after overexpression of SLC12A5 were detected. (C) Knockdown of SLC12A5 by siRNAs were identified by qRT-PCR and western blot in PC3 cells. (D) Colony formation of PC3 cells after knockdown of SLC12A5 were detected. (E) Cell proliferation curves were detected by CCK-8 assays after knockdown of SLC12A5 in PC3 cells. (F) Migration of PC3 cells after knockdown of SLC12A5 were detected by transwell assays. Magnification, ×200. *, P < 0.05; **, P < 0.01.


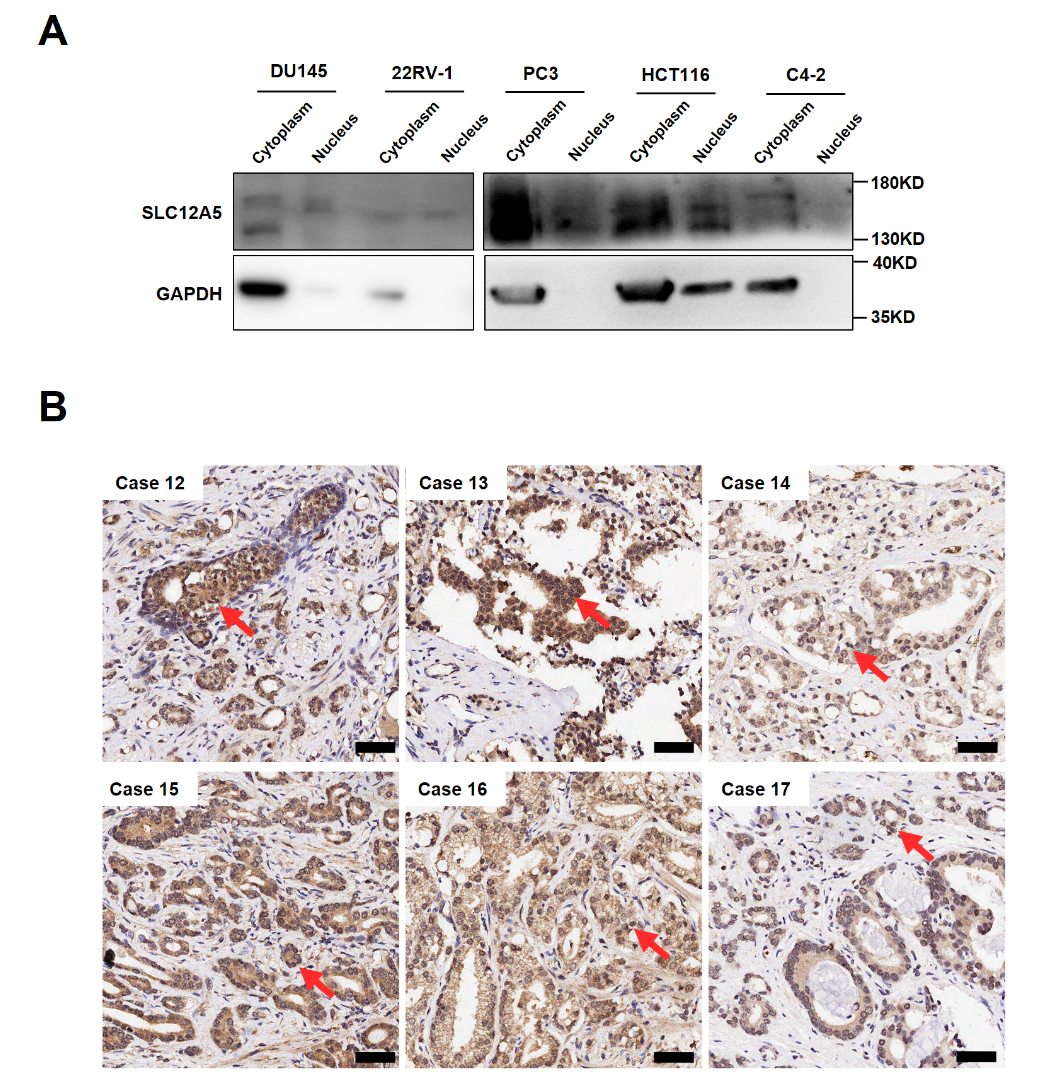


**Supplementary Figure S3. SLC12A5 protein can be detected in the cell nucleus.** (A) Western blot detection of the subcellular localization of SLC12A5 protein in prostate cancer cells. HCT116 cell line was used as a positive nuclear localization control and C4-2 cell line was used as a negative nuclear localization control. GAPDH served as a cytoplasmic localization control. (B) Immunohistochemical staining of prostate cancer tumor tissues (Case 12-Case 17) showed SLC12A5 protein was partly localized in the nucleus. Scale bars represent 50 μm.


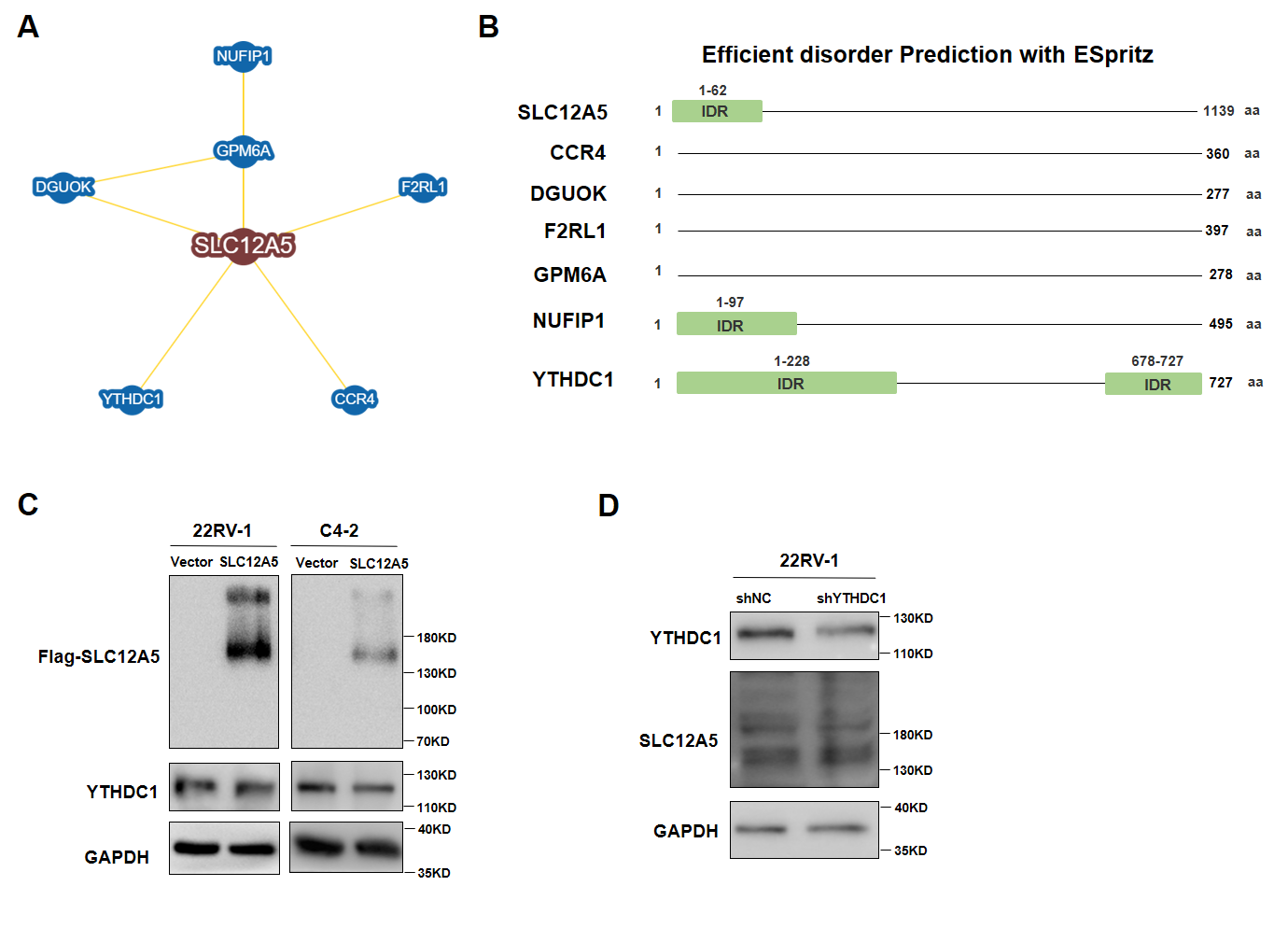


**Supplementary Figure S4. SLC12A5 protein binds to YTHDC1 but does not affects the protein levels of YTHDC1.** (A) The entire protein interaction network of SLC12A5 interacting proteins was plot using BioGRID analysis. (B) The intrinsically disordered region (IDR) is predicted using ESpritz. (C) YTHDC1 protein levels were detected by western blot when SLC12A5 overexpression in 22RV-1 and C4-2 cells. (D) SLC12A5 protein levels were detected by western blot when knocking down of YTHDC1 in 22RV-1 cells.


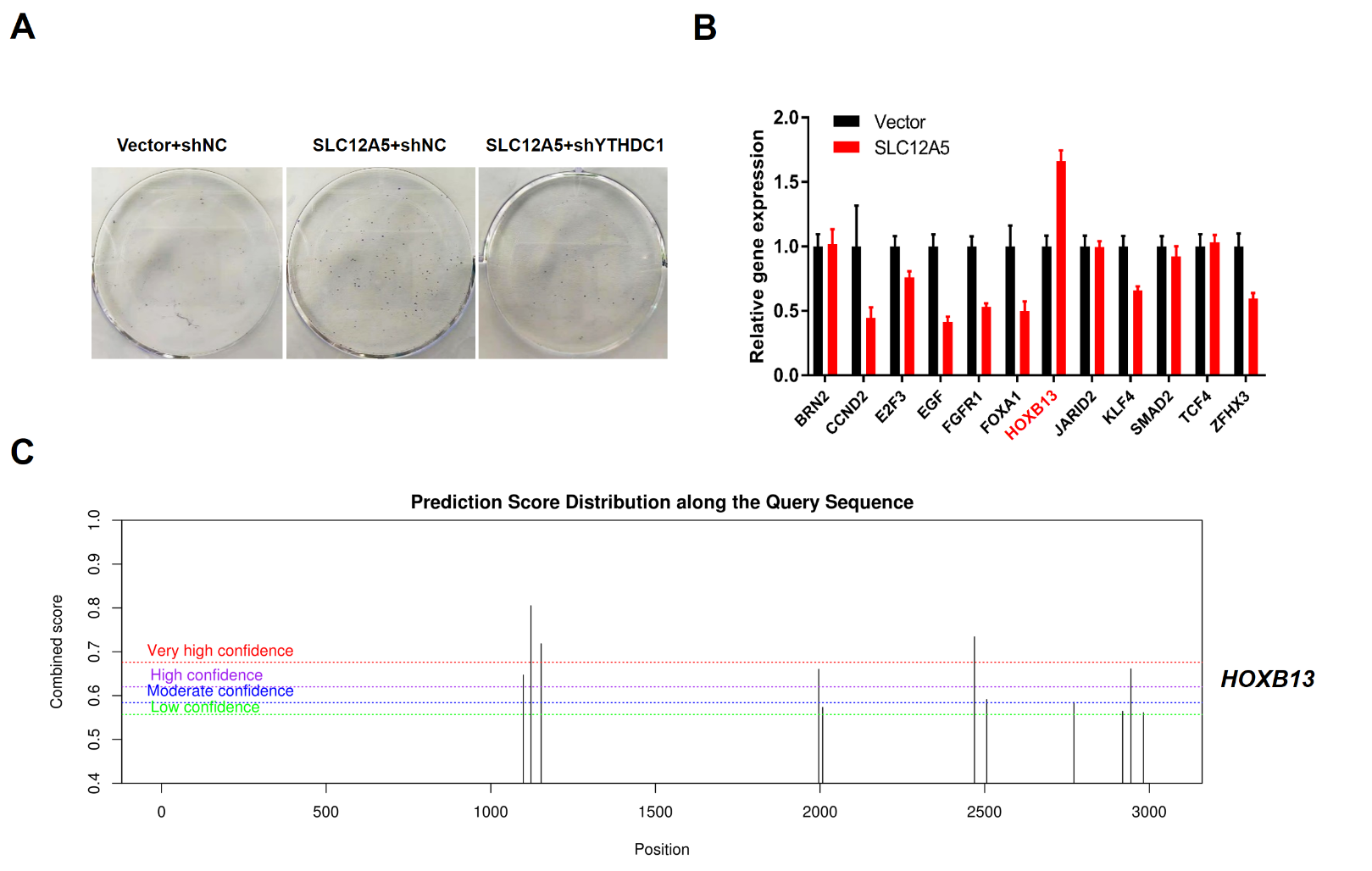


**Supplementary Fig. S5. SLC12A5 plays a tumor-promoting role partially depending on YTHDC1 and HOXB13.** (A) Colony formation assay was conducted to clarify the rescue effect of YTHDC1 on the cell proliferation in 22RV-1 cells. (B) The expression levels of 12 genes, which were essential for prostate cancer progression and neuroendocrine differentiation, were detected by qRT-PCR after overexpression of SLC12A5 in 22RV-1 cells. (C) The m^6^A RNA modification sites in *HOXB13* mRNA sequence identified by using SRAMP prediction server (http://www.cuilab.cn/sramp).
