# Supplementary material for: A potassium-chloride co-transporter promotes tumor progression and castration resistance of prostate cancer through m6A reader YTHDC1
Source: Cell Death Dis. 2023 Jan 6;14(1):7. doi: 10.1038/s41419-022-05544-8 (PMC9822915; doi:10.1038/s41419-022-05544-8)

Figure 2A

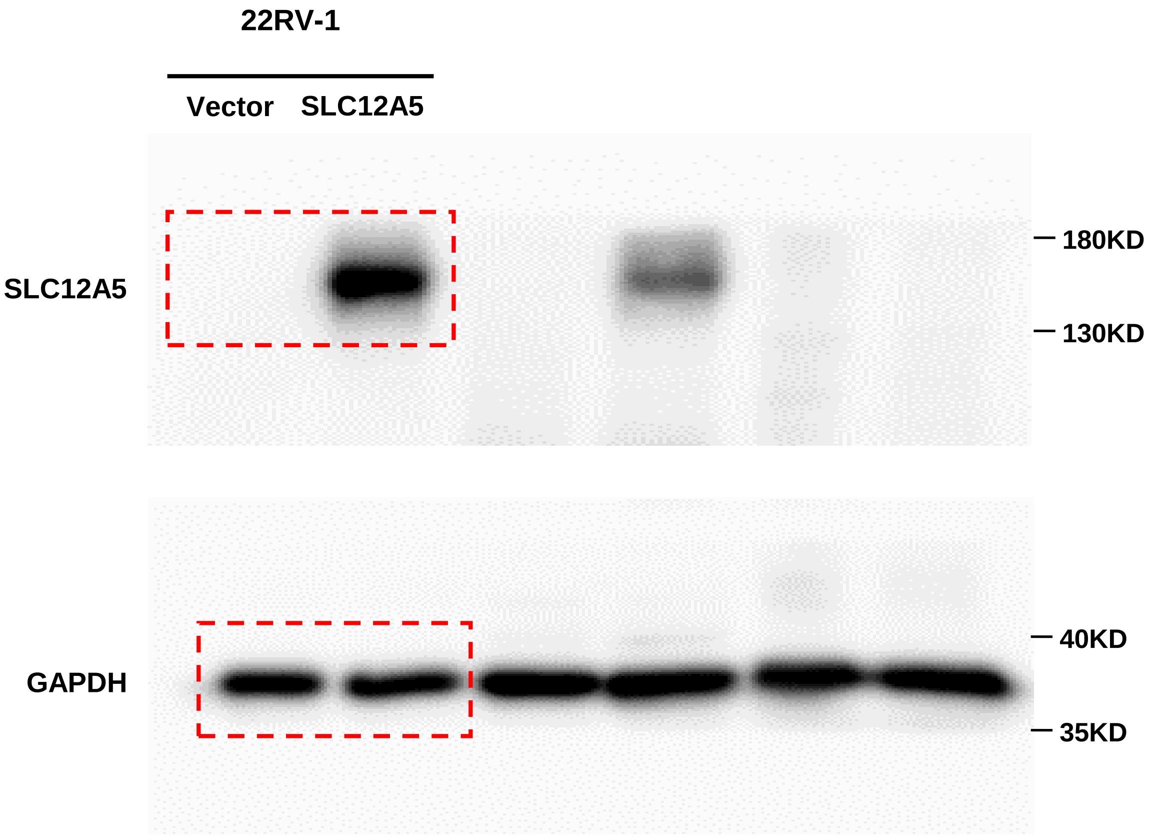

Figure 2E

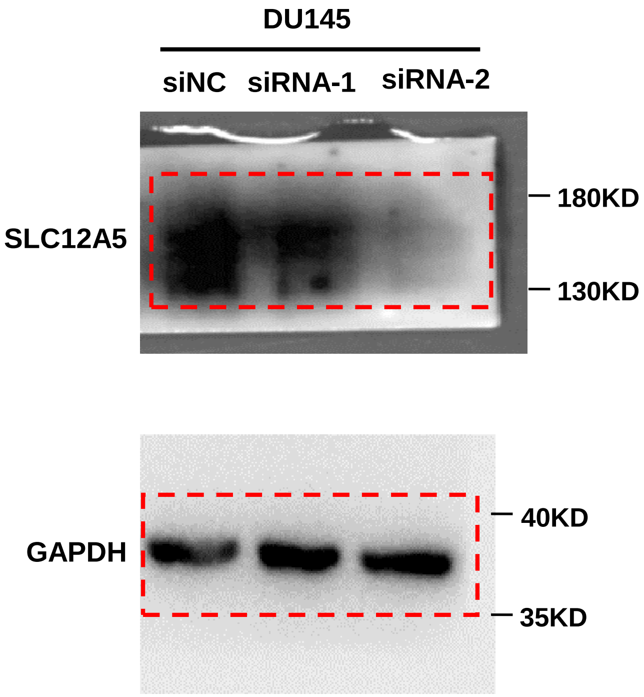

Figure 5B

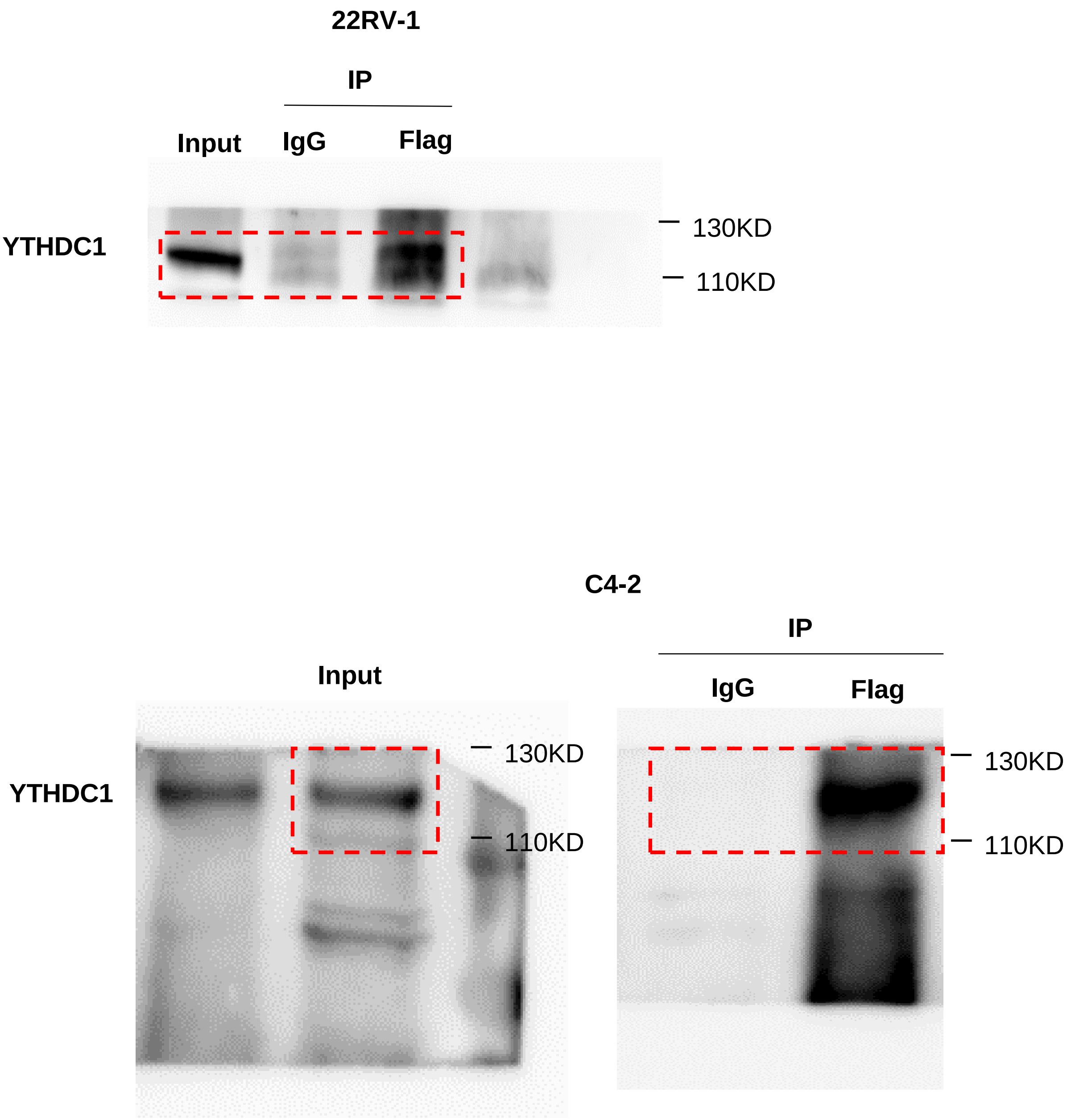
22RV-1
IP
Input
IgG
Flag
YTHDC1
130KD
110KD
C4-2
IP
Input
IgG
Flag
YTHDC1
130KD
110KD
130KD
110KD

Figure 6A

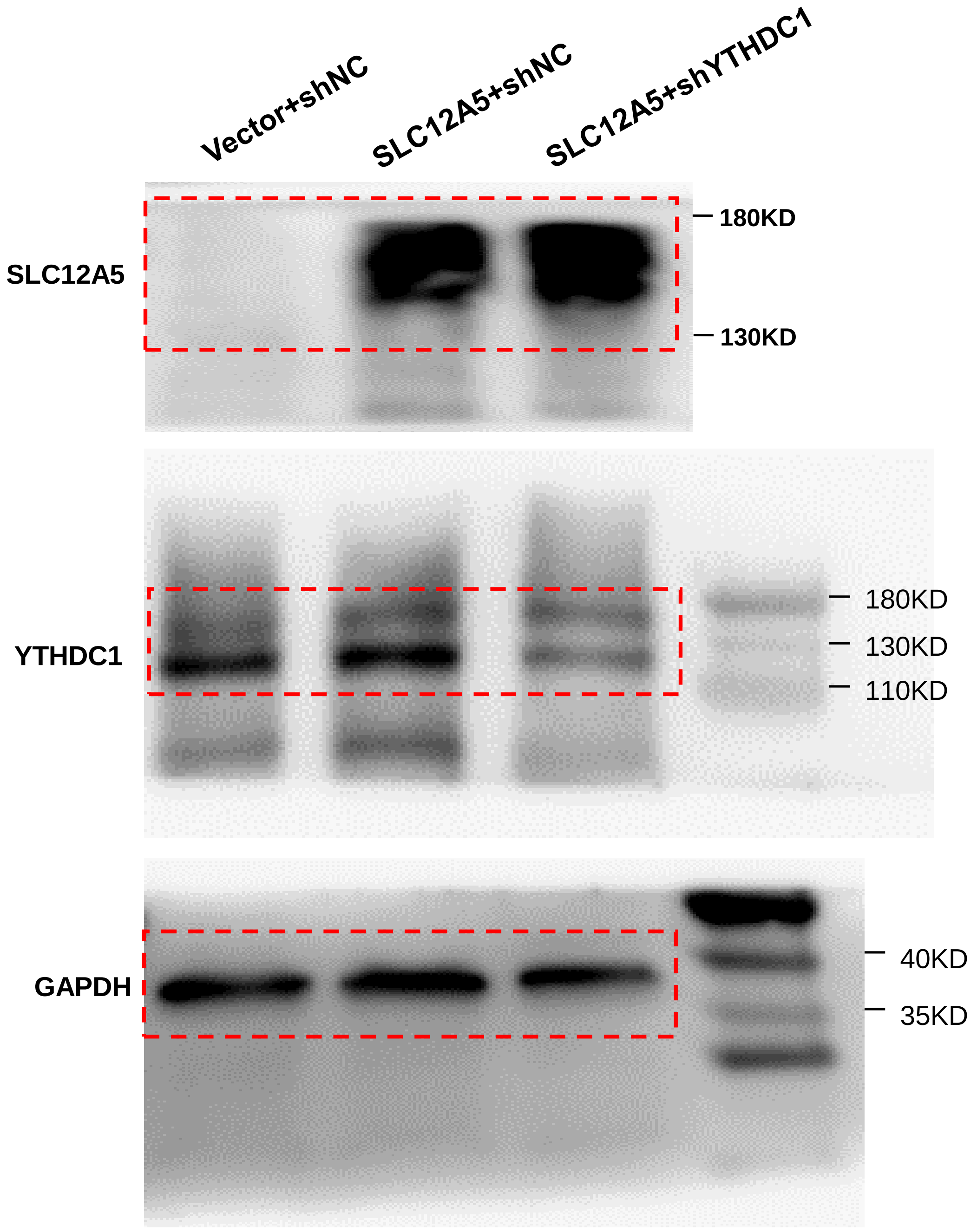
Vector+shNC
SLC12A5+shNC
SLC12A5+shYTHDC1
SLC12A5
180KD
130KD
YTHDC1
180KD
130KD
110KD
GAPDH
40KD
35KD

Figure 6E

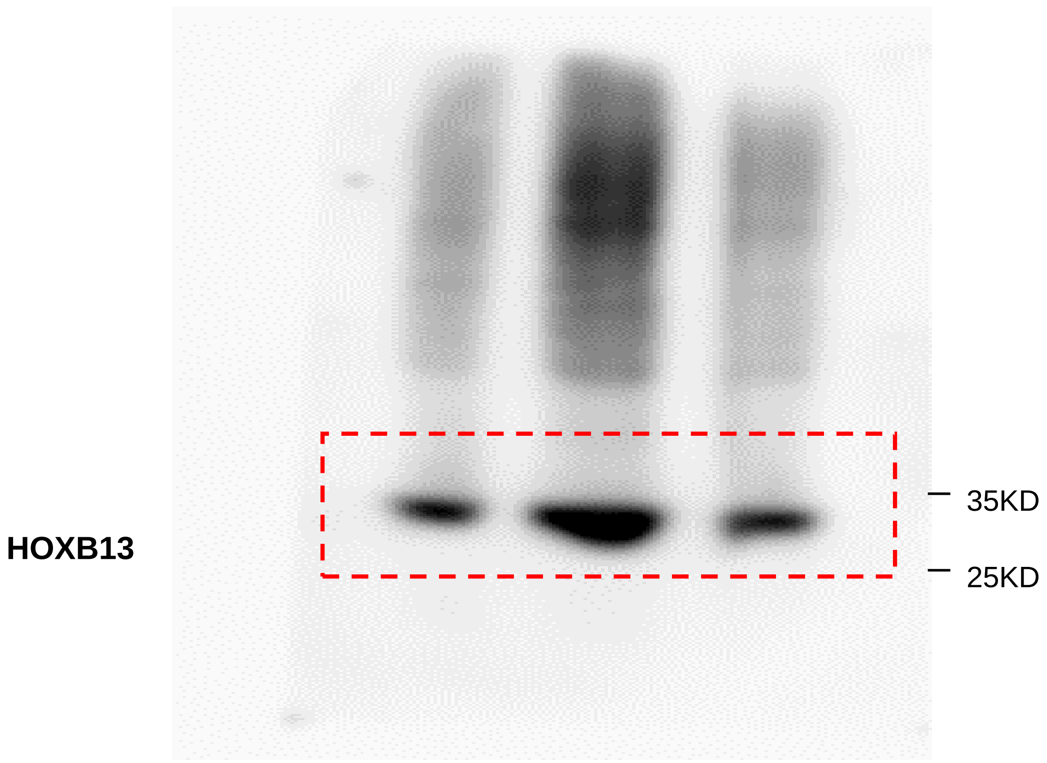

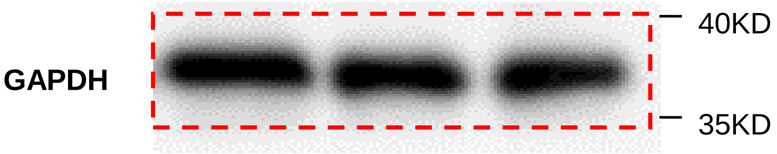

Supplementary Figure S2A

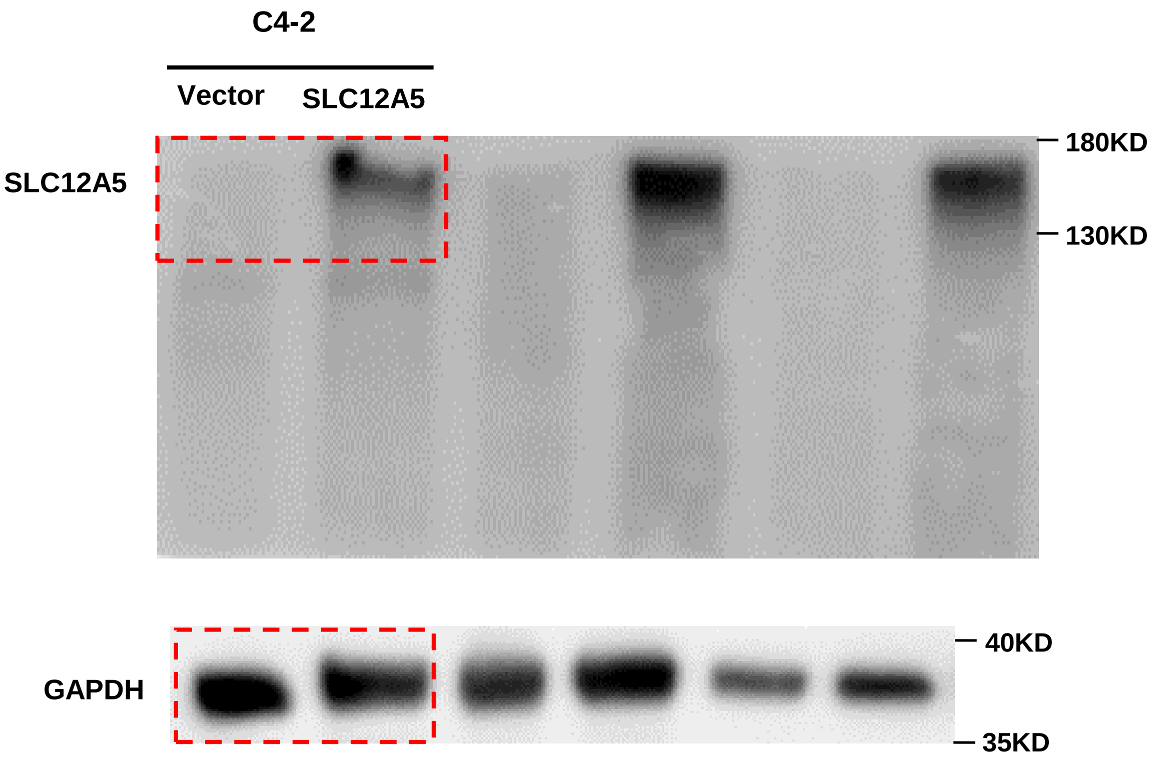

Supplementary Figure S2C

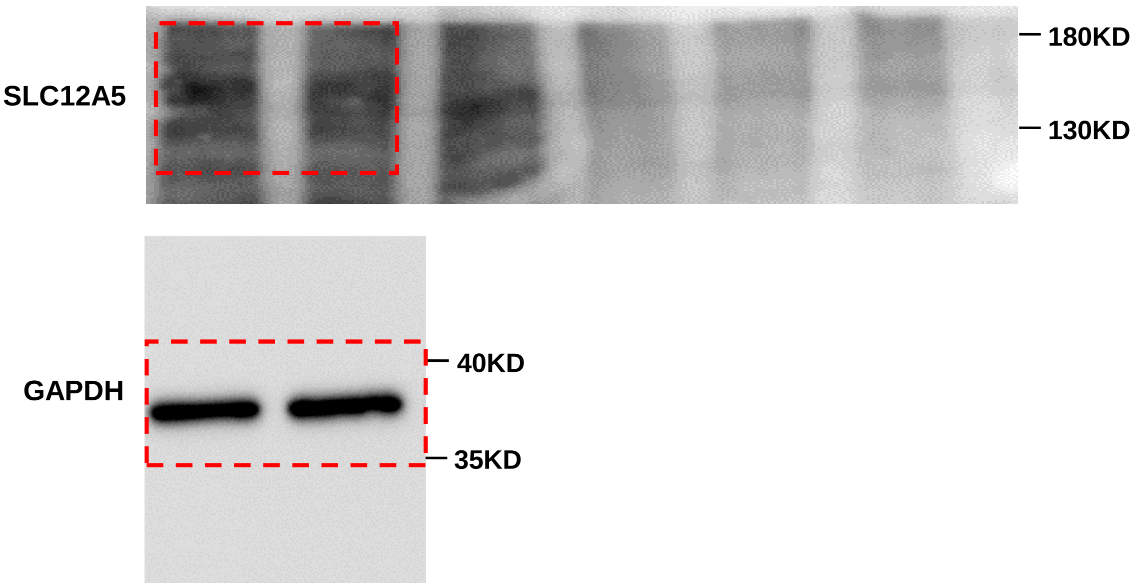

Supplementary Figure S3A

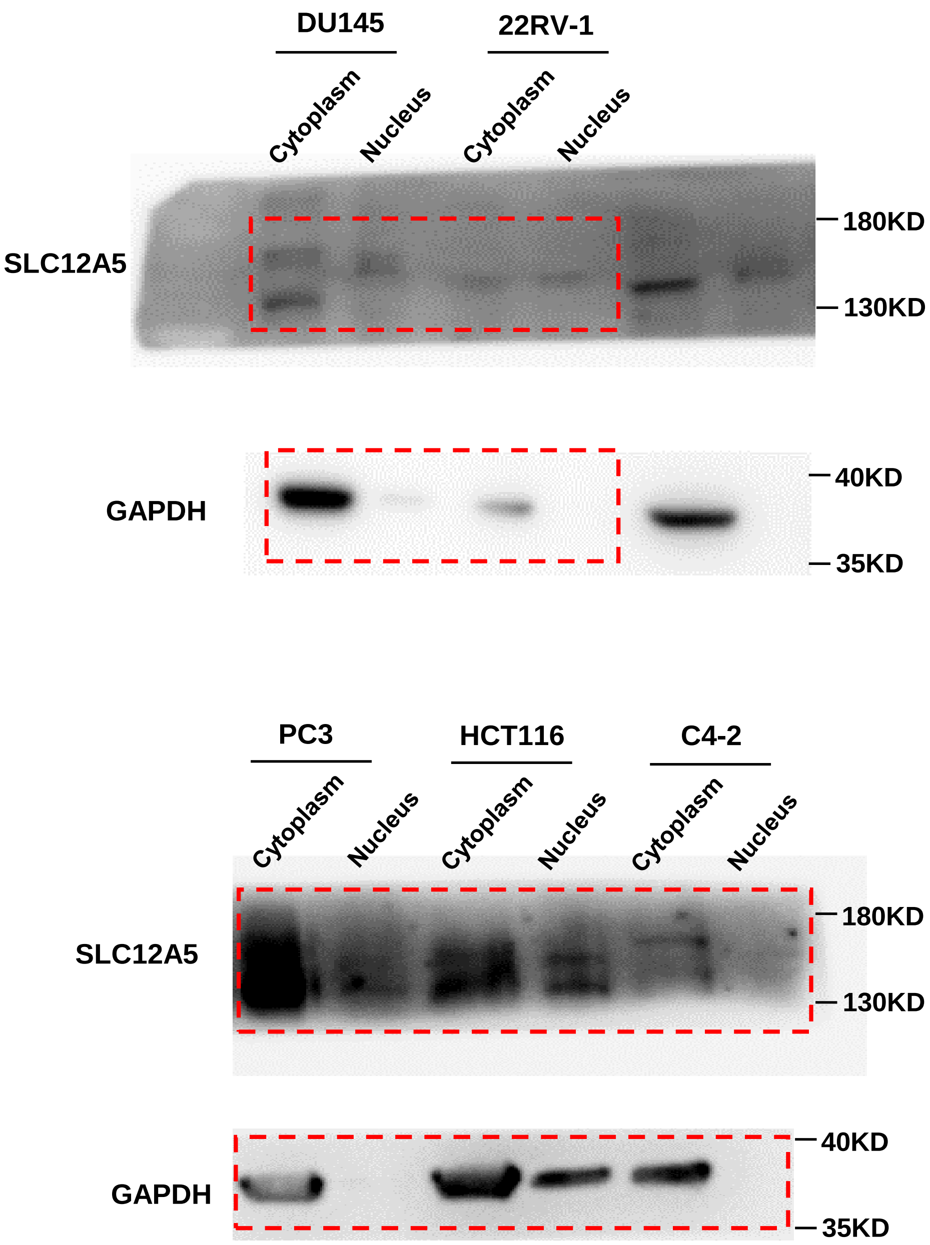

Supplementary Figure S4C

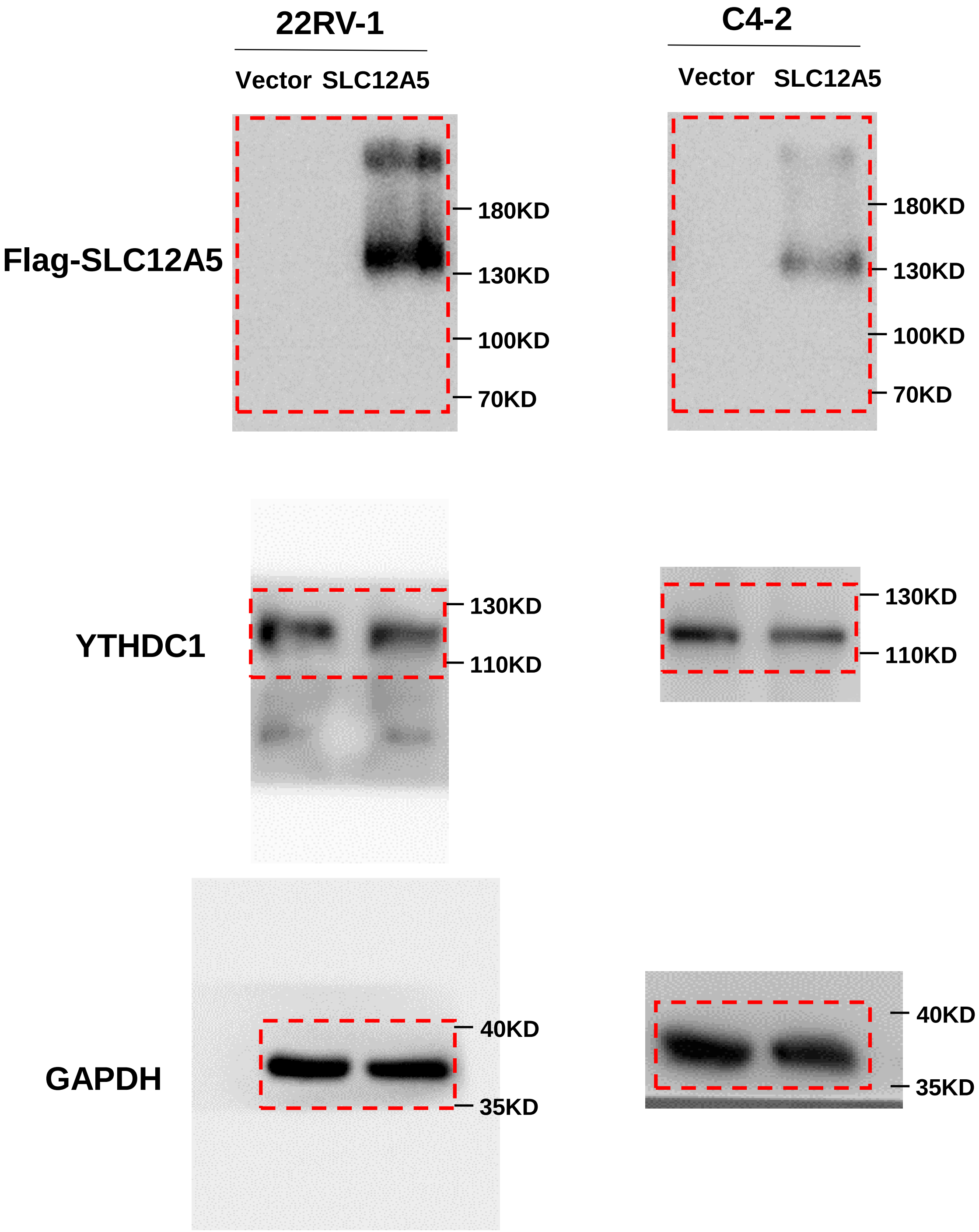

Supplementary Figure S4D

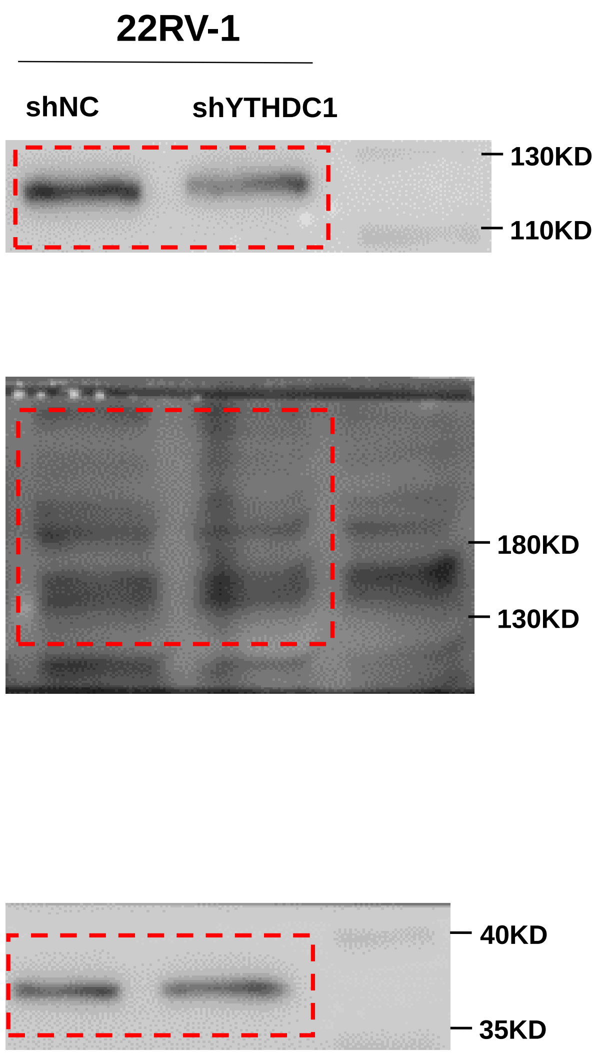

Supplement: Supplementary file 2 — Original uncropped Western Blots [file 41419_2022_5544_MOESM2_ESM.pdf]
